# The meta-gut: community coalescence of animal gut and environmental microbiomes

**DOI:** 10.1038/s41598-021-02349-1

**Published:** 2021-11-30

**Authors:** Christopher L. Dutton, Amanda L. Subalusky, Alvaro Sanchez, Sylvie Estrela, Nanxi Lu, Stephen K. Hamilton, Laban Njoroge, Emma J. Rosi, David M. Post

**Affiliations:** 1 Department of Ecology and Evolutionary Biology, Yale University, 165 Prospect St., New Haven, CT 06511 USA; 2 Department of Biology, University of Florida, Gainesville, FL USA; 3 Microbial Sciences Institute, Yale University, New Haven, CT USA; 4 W.K. Kellogg Biological Station and Department of Integrative Biology, Michigan State University, Hickory Corners, MI USA; 5 Cary Institute of Ecosystem Studies, Millbrook, NY USA; 6 National Museums of Kenya, Nairobi, Kenya

**Keywords:** Ecosystem ecology, Limnology

## Abstract

All animals carry specialized microbiomes, and their gut microbiota are continuously released into the environment through excretion of waste. Here we propose the *meta-gut* as a novel conceptual framework that addresses the ability of the gut microbiome released from an animal to function outside the host and alter biogeochemical processes mediated by microbes. We demonstrate this dynamic in the hippopotamus (hippo) and the pools they inhabit. We used natural field gradients and experimental approaches to examine fecal and pool water microbial communities and aquatic biogeochemistry across a range of hippo inputs. Sequencing using 16S RNA methods revealed community coalescence between hippo gut microbiomes and the active microbial communities in hippo pools that received high inputs of hippo feces. The shared microbiome between the hippo gut and the waters into which they excrete constitutes a *meta-gut* system that could influence the biogeochemistry of recipient ecosystems and provide a reservoir of gut microbiomes that could influence other hosts. We propose that *meta-gut* dynamics may also occur where other animal species congregate in high densities, particularly in aquatic environments.

## Introduction

Animals alter the functioning of ecosystems by consuming plant and animal matter, through the transport and excretion of nutrients, and in myriad other ways^1–6^. In many cases, the activities of animals directly or indirectly influence microorganisms (microbes), which in turn regulate the major biogeochemical cycles, although such linkages remain to be fully investigated^7–9^. Abundant evidence exists for altered decomposition and nutrient cycling in response to organic matter and nutrient excretion and egestion by animals, but few studies have explicitly examined the role of the externally released animal gut microbiome in mediating those changes^5,6,10–13^, although see^14^.

Community coalescence theory is an ecological framework for investigating the mixing of entire microbial communities and their surrounding environments^15–17^, but much of the focus has been on metacommunity dynamics among the microbiota, with less attention on the ecosystem implications of resource flows that accompany this mixing^18^. Animal excretion and egestion present a unique case of community coalescence by effectively mixing the animal gut microbiome with preexisting microbial communities, together with organic matter, nutrients, and metabolic byproducts that may also be excreted or egested. Heavy rates of such loading can shape the external environment in ways that support the persistence of gut microbiota outside the host gut, particularly in aquatic ecosystems, increasing the likelihood that ex situ gut microbes could influence ecosystem processes and be re-ingested by other consumers. We propose that the resulting *meta-gut* system is a dynamic interplay of abiotic resources and microbial communities between the host gut, the external environment, and possibly the guts of other individual hosts that inhabit the same environment.

**Figure 1:**
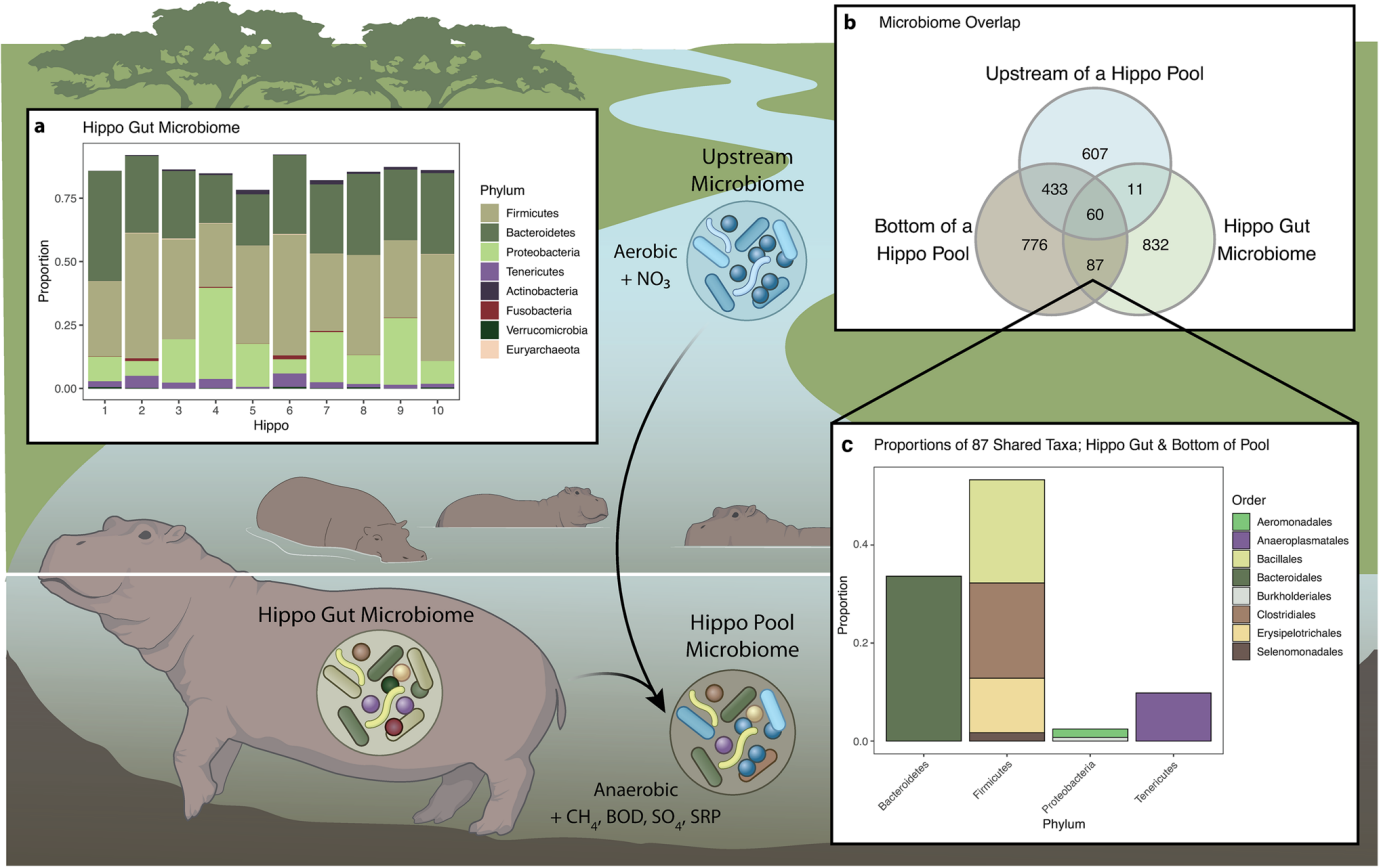
The coalescence of environmental and gut microbial communities to form the *meta-gut* system in a hippo pool. **(a)** Proportions of the 8 most abundant phyla from the active microbial communities present in the guts of 10 individual hippos (genus provided in Supplementary Fig. S2). The hippo gut microbiome was dominated by Firmicutes, Bacteroidetes and Proteobacteria. Less abundant phyla are not shown. **(b)** Venn diagram showing the overlap of active microbial taxa between the hippo gut microbiome (N = 10 samples), aquatic microbial communities upstream of high-subsidy hippo pools (N = 19) and those from the bottom waters of high-subsidy hippo pools (N = 19). **(c)** Phylum and order for the 87 active taxa from the 8 most abundant orders shared between the hippo gut microbiome and the bottom waters of high-subsidy hippo pools (genus provided in Supplementary Table S1).

Hippos (*Hippopotamus amphibius*) have profound effects on aquatic ecosystems in which they wallow during the day by adding large amounts of organic matter and nutrients from their nighttime terrestrial grazing via defecation and urination^19–23^, and their fecal inputs are accompanied by abundant enteric microbes (Fig. 1a). These resource subsidies interact with environmental characteristics of the recipient ecosystem to alter ecosystem function^6,23,24^. The organic matter excreted by hippos accumulates at the bottom of hippo pools under low to moderate discharge, and in pools with high hippo densities the decomposition of this organic matter often depletes dissolved oxygen in the water column^23^. As hippo pools become anoxic, the pool environment becomes more similar to the hippo gut, increasing the likelihood that some of the enteric microbes will survive and even function outside the host gut. Anoxia is a persistent state in the bottom waters of many hippo pools until high flow events flush organic matter downstream and reaerate the water column^23,25^, briefly exposing the microbial community to oxic conditions before organic matter loading by hippos once again drives pools towards anoxia. The conditions produced by hippos provide a unique opportunity to investigate the importance of the *meta-gut* system.

The Mara River flows through the Serengeti Mara Ecosystem in Kenya and Tanzania (see Supplementary Fig. S1). There are over 4000 hippos distributed among approximately 170 hippo pools in the Kenyan portion of the Mara River and its seasonal tributaries^26^. Water is present in hippo pools year-round even in the seasonal tributaries, some of which may exhibit very low or no flow during dry periods. Hippo numbers and water residence times of hippo pools (pool volume/discharge) interact to shape in-pool and downstream biogeochemistry in the Mara River system^23^. We classified pools by the magnitude of subsidy inputs: high-, medium-, and low-subsidy pools. Low-subsidy pools remain oxic, while high-subsidy pools are typically anoxic, except during periodic flushing flows.

Here we examine the *meta-gut* phenomenon in hippo pools by sequencing the microbial communities of hippo guts and hippo pools in the field across a range of environmental conditions, and we consider the biogeochemical implications of the *meta-gut* system. We also conducted a microcosm experiment to investigate the role of both microbiomes and viruses (specifically bacteriophages) from the hippo gut in driving biogeochemical processes and microbial community changes within hippo pools.

## Results

### Community changes over space

We characterized the microbial communities in the hippo gut by collecting fresh hippo feces from multiple individuals across multiple pools on the landscape. There was little variability in the structure of the hippo gut microbiome among the samples from 10 individuals, and the microbiomes of all samples were dominated by Firmicutes, Bacteroidetes, Proteobacteria and Tenericutes (Fig. 1a). Dominant genera of the hippo gut microbiome include *Macellibacteroides* (14% average across all samples), *Acinetobacter* (5%), *Bacteroides* (7%), and *Lysinibacillus* (5%) (Supplementary Fig. S2). The gut microbiomes of hippos sharing a high-subsidy pool were not distinct relative to those of hippos from other high-subsidy pools and had similar dispersions (PERMANOVA: F = 0.68524, P = 0.886, PERMDISP: F = 7.3918, P = 0.013, Supplementary Fig. S3).

**Figure 2:**
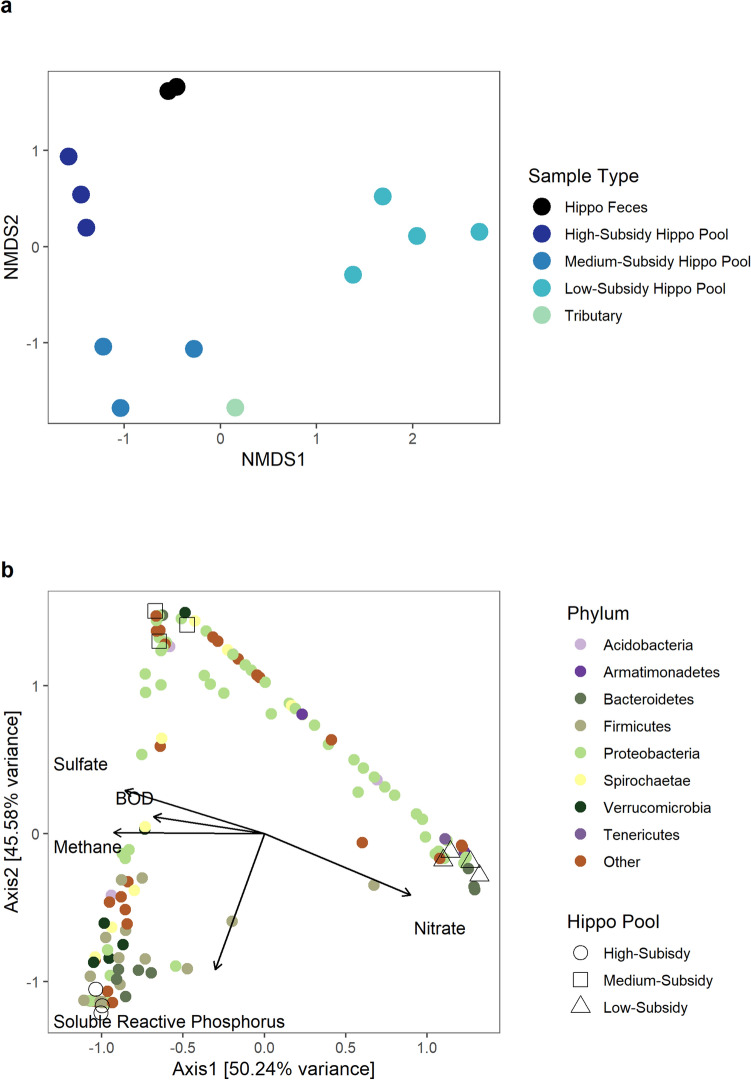
**(a)** NMDS ordination (NMDS Stress = 0.1) of the Bray–Curtis dissimilarity matrix for the active aquatic microbial communities in hippo feces (N = 2), in the tributary water (N = 1), and in the bottom waters of high-subsidy (N = 3), medium-subsidy (N = 3), and low-subsidy hippo pools (N = 4). **(b)** Canonical correspondence analysis (CCA) of the active aquatic microbial communities (top 8 phyla are represented as colored circles with all other phyla represented as a brown circle) constrained by five environmental variables from bottom waters at the three types of sample sites: high-subsidy (hollow circle, N = 3), medium-subsidy (hollow triangle, N = 3), and low-subsidy hippo pools (hollow square, N = 4).

We characterized the microbial communities in the water column of hippo pools across a gradient of hippo subsidy. Aquatic microbial communities in the bottom waters of high-, medium-, and low-subsidy hippo pools were distinctly different from one another (PERMANOVA: F = 3.3146, P = 0.002). They also had different dispersions (PERMDISP: F = 0.3532, P = 0.72); however, their differences in diversity were supported through clustering within a NMDS ordination (NMDS Stress = 0.1, Fig. 2a). The microbial communities in high-subsidy pools were more similar to those of the hippo gut microbiome than to the aquatic microbial community sampled from an area outside the influence of hippos (tributary, Fig. 2a).

We observed large differences between the three types of hippo pools when constrained by microbial communities and biogeochemistry (PERMANOVA: F = 1.3641, P = 0.011). High-, medium-, and low-subsidy hippo pools were strongly separated by the CCA ordination, which accounted for approximately 90% of the variability in the active microbial community structure within the first two axes of the constrained ordination (Fig. 2b). We observed strong relationships between the microbial community and biogeochemical constituents affected by microbial metabolism. Concentrations of dissolved methane, sulfate, and BOD were correlated and had effects (influence) that were opposite from nitrate, all of which were strongly related to axis 1 (Fig. 2b). Soluble reactive phosphorus (SRP) was strongly related to axis 2. Low-subsidy hippo pools were strongly associated with higher concentrations of nitrate. Medium-subsidy hippo pools were strongly associated with higher concentrations of sulfate, methane and BOD, but low levels of SRP. High-subsidy hippo pools were strongly associated with higher concentrations of methane, BOD, sulfate and SRP. Firmicutes were more closely associated with high- subsidy pools and higher concentrations of methane, BOD, and SRP.

**Figure 3:**
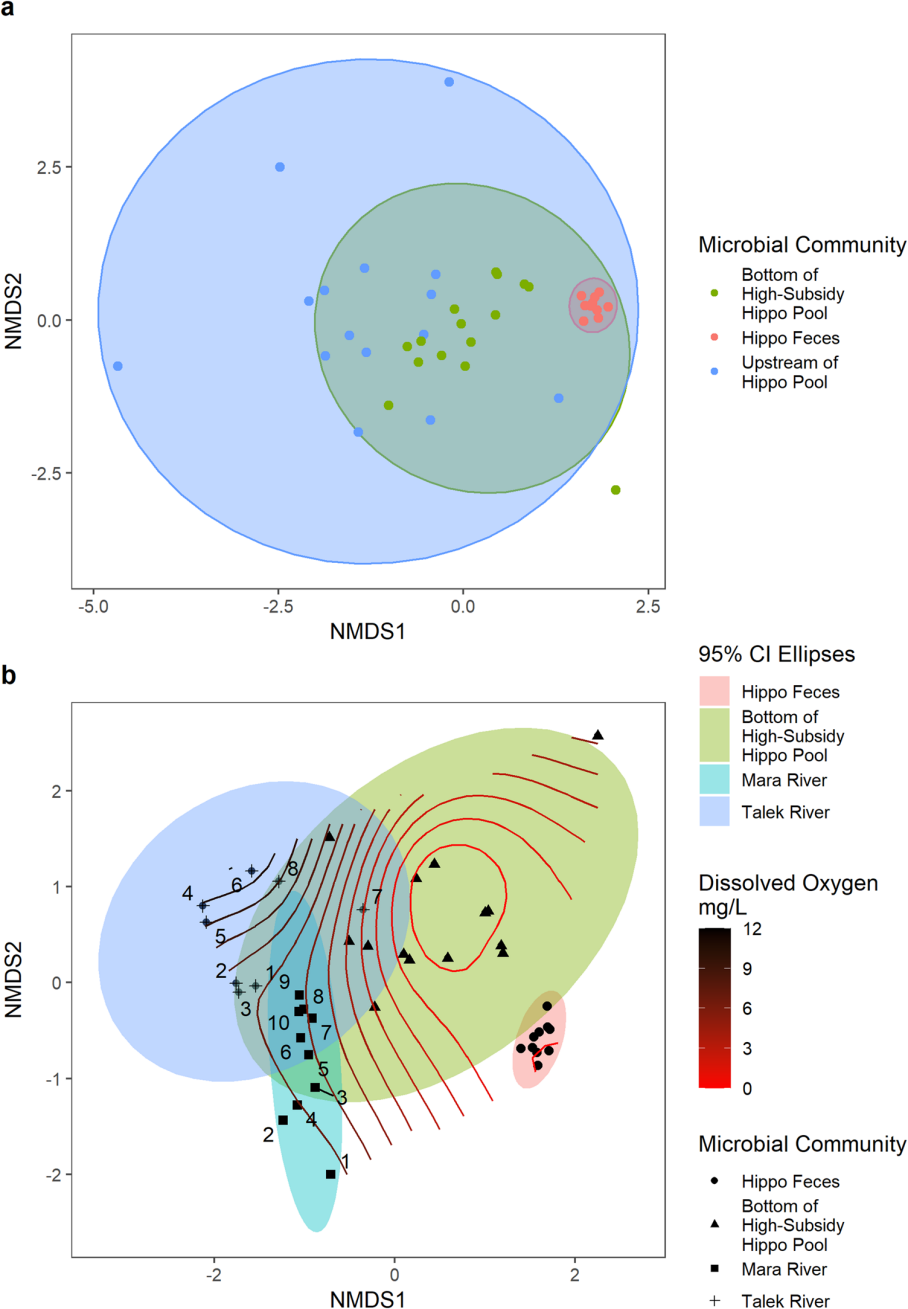
NMDS ordination of the Bray–Curtis dissimilarity matrix for the active microbial communities. **(a)** Hippo feces (N = 10), river waters upstream of high-subsidy hippo pools (N = 15), and the bottom waters of high-subsidy hippo pools (N = 15). 95% confidence ellipses are provided. NMDS Stress 0.12. **(b)** Hippo feces (N = 10), the bottom waters of high-subsidy hippo pools (N = 15), and water samples collected along a transect of the Mara (N = 10) and Talek (N = 8) rivers. Numbers indicate the sampling site along the transect starting at 1 and moving downstream in ascending order. Lines represent the dissolved oxygen gradient separating the sites. 95% confidence ellipses are provided. NMDS Stress 0.12.

We characterized the microbial communities in the water column upstream of and within hippo pools, and along a gradient of hippo density in the Mara River and its tributary, the Talek River. Aquatic microbial communities upstream of hippo pools were more variable than the communities found in the bottom waters within hippo pools (Fig. 3a). Active microbial communities in the Mara and Talek rivers were similar to one another and dissimilar to communities in hippo feces (Fig. 3b). Active microbial communities in the bottom waters of high-subsidy hippo pools were similar to both the riverine communities and the hippo feces communities. Along the upstream (site 1) to downstream (site 10) gradient of hippo influence found in the Mara River, the Mara River microbial community shifted upward on the ordination towards the microbial communities found in the Talek and in the bottom water of high-subsidy hippo pools. DO was lowest in hippo feces and the bottom of high-subsidy hippo pools. DO was highest in the middle reaches of the Talek river.

We compared the microbial taxa collected from upstream, downstream, on the surface and on the bottom of a set of different hippo pools. The class Alphaproteobacteria were more abundant upstream of hippo pools compared to any other location (Supplementary Figs. S5, S6). The genus *Dechloromonas* was more abundant downstream of hippo pools. The phylum Chloroflexi, the class Bacteroidia, the orders Bacteroidales and Desulfobacterales, the families Desulfobulbaceae and Porphyromonadaceae, and the genera *Desulfobulbus* and *Macellibacteroides*, were all more abundant at the bottom of hippo pools compared to other locations.

Eighty-seven actively functioning taxa were shared between the hippo gut microbiome (hippo feces) and the bottom waters of high-subsidy hippo pools, but not found immediately upstream of high-subsidy hippo pools (Fig. 1b). The comparatively larger overlap exhibited between the bottom of the hippo pool and the upstream sites may be due to the presence of other hippo pools located 20–100 m upstream. Most of those taxa are from the phyla Firmicutes and Bacteroidetes and are commonly found in the intestines of animals (Fig. 1c, Supplementary Table S1). Notably, *Clostridia*, obligate anaerobes, were responsible for approximately 19% of the overlap between the active microbial communities in the bottom of the hippo pools and the hippo gut microbiome. *Macellibacteroides*, an obligate anaerobe, was responsible for approximately 16% of the overlap, and was previously described from anaerobic wastes from an abattoir in Tunisia^27^. *Prevotellaceae*, commonly found in the intestines of animals and which helps break down proteins and carbohydrates, was responsible for 11% of the overlap^28^.

We compared the microbial taxa in and around hippo pools with the biogeochemistry of their environment and found that taxa associated with the hippo gut microbiome (such as the genus *Macellibacteroides* and the phylum Firmicutes) were significantly correlated to higher BOD, H_2_S, Fe(II), DOC, CO_2_, CH_4_, K^+^, SRP, TN, TP, NH_4_^+^-N, conductivity, Mg^2+^, and Ca^2+^, and negatively correlated to dissolved oxygen, NO_3_^−^ and SO_4_^2−^ (Supplementary Fig. S7). Taxa associated with environmental reservoirs (such as the families Anaerolineaceae and Bdellovibrionaceae and the genus *Novospingobium*) did not have as many significant correlations but tended to have opposite associations to biogeochemistry compared to the hippo gut microbiome associated taxa. The class Alphaproteobacteria was significantly correlated with higher NO_3_^−^, SO_4_^2−^ and oxidation redox potential and negatively correlated with NH_4_^+^-N, CO_2_, CH_4_, Fe(II), DOC, BOD, TN, TP, Mg^2+^, Ca^2+^ and K^+^.

### Community changes over time

**Figure 4:**
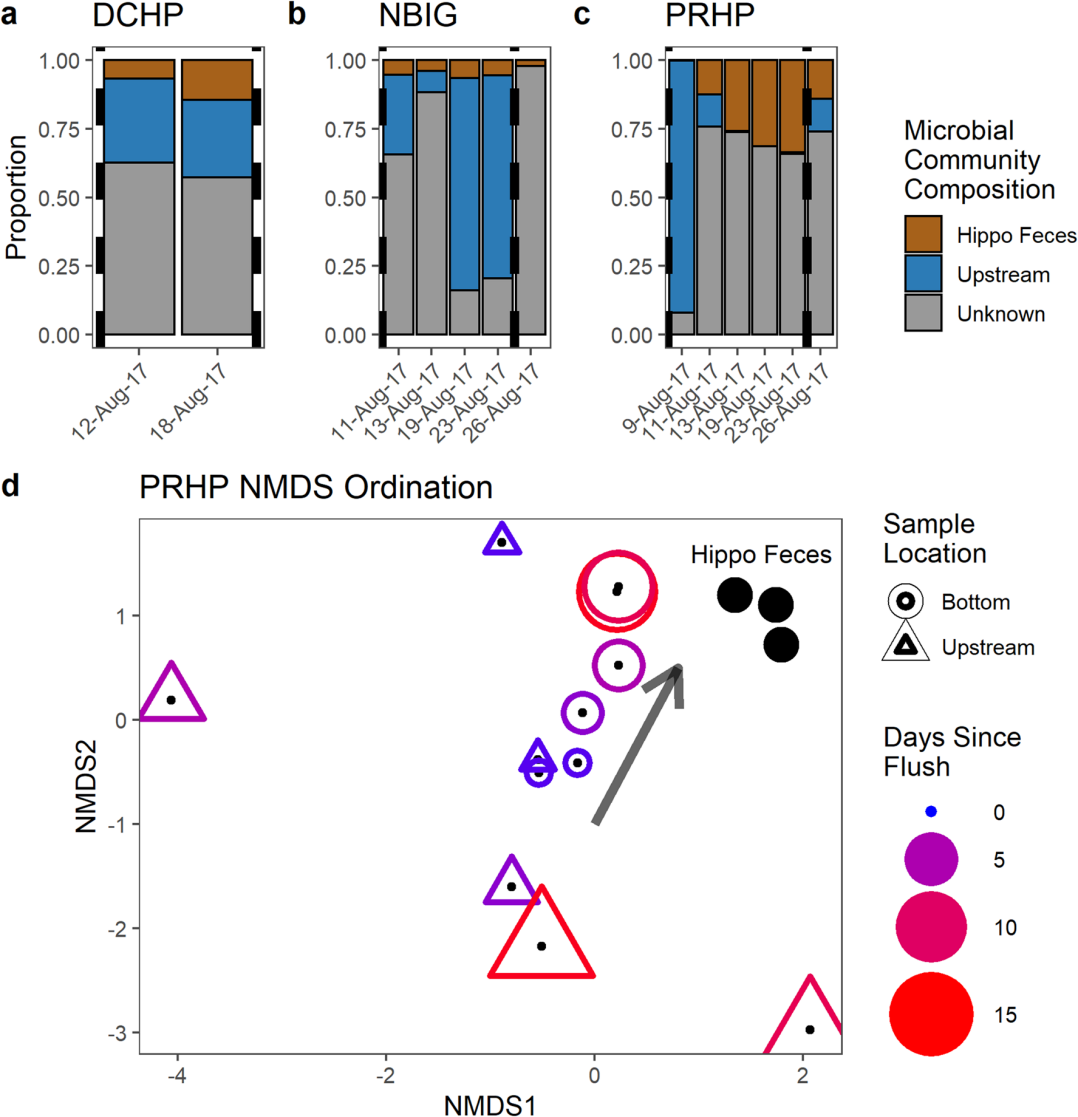
The proportion of the active microbial communities in the bottom water of high-subsidy hippo pools from hippo feces, upstream river waters, or unknown origin in **(a)** DCHP Hippo Pool, **(b)** NBIG Hippo Pool and, **(c)** PRHP Hippo Pool. Flushing flows are represented by a dashed vertical line. The first flushing flow occurred on 8-Aug-17. The second flushing flow occurred on 25-Aug-17. **(d)** NMDS of the Bray–Curtis Dissimilatory matrix of the active microbial communities for hippo feces and the upstream and bottom samples from the PRHP high-subsidy hippo pool during the flushing events. Hippo microbiome is represented as filled black circles in the upper right of the ordination. Samples from the bottom waters are notated as a circle. Samples from the upstream are notated as a triangle. Color gradient from blue to red and size gradient from small to large represent the days since the last flushing flow. Immediately after the flushing flow, the upstream and bottom microbial communities looked similar. As time went on, the microbial community in the bottom waters looked more similar to the hippo feces microbiome than the upstream community (movement denoted as an arrow).

We characterized the microbial communities in the water column of three high-subsidy hippo pools during transitions between aerobic and anaerobic states in response to flushing flows. As pools transitioned between anoxic and oxic states, we observed substantial changes in the proportional contribution of sources of the aquatic microbial community. In the bottom waters of all three pools, the proportion of the aquatic microbial community from the hippo gut increased over the interval between the first flushing event (7 August) and the next flushing event (25 August). The increase was most pronounced in PRHP, which had over 30% of the active aquatic microbial community derived from hippo feces just prior to the August 25th flushing event (Fig. 4c). *Macellibacteroides* and *Odoribacter* comprised the highest proportion of any of the taxa from the hippo gut that continued to function in the pool, and their proportions increased over time between flushing flows (Supplementary Fig. S8). Aquatic microbial communities in the bottom waters of hippo pools were more similar to those of upstream waters immediately after the flushing flow, then diverged as flows receded (Fig. 4d, Supplementary Fig. S9). In intervals between flushing flows, the aquatic microbial communities from the bottom waters of the hippo pools became more similar to hippo gut communities until the next flushing flow occurred. Across all pools, the majority of the microbial taxa could not be attributed to either hippo feces or upstream sources, which may indicate the growth of free-living microbial populations adapted to hypoxic or anoxic conditions (Fig. 4, Supplementary Fig. S10).

### Influence of hippo gut microbiome on biogeochemistry in a mesocosm experiment

We conducted a mesocosm experiment to understand the changes in microbial communities that occur as a hippo pool goes anoxic, and to test the role of microbial taxa associated with the hippo gut in driving biogeochemical changes within the hippo pools. An additional goal was to test the impact of fecal bacteriophages from the hippo gut on the microbial communities (which are composed almost entirely of bacteria) and the biogeochemical processes they mediate in hippo pools. We used a destructive sampling design with control (sterilized), bacteria, and bacteria + virus treatments (Supplementary Fig. S4).

**Figure 5:**
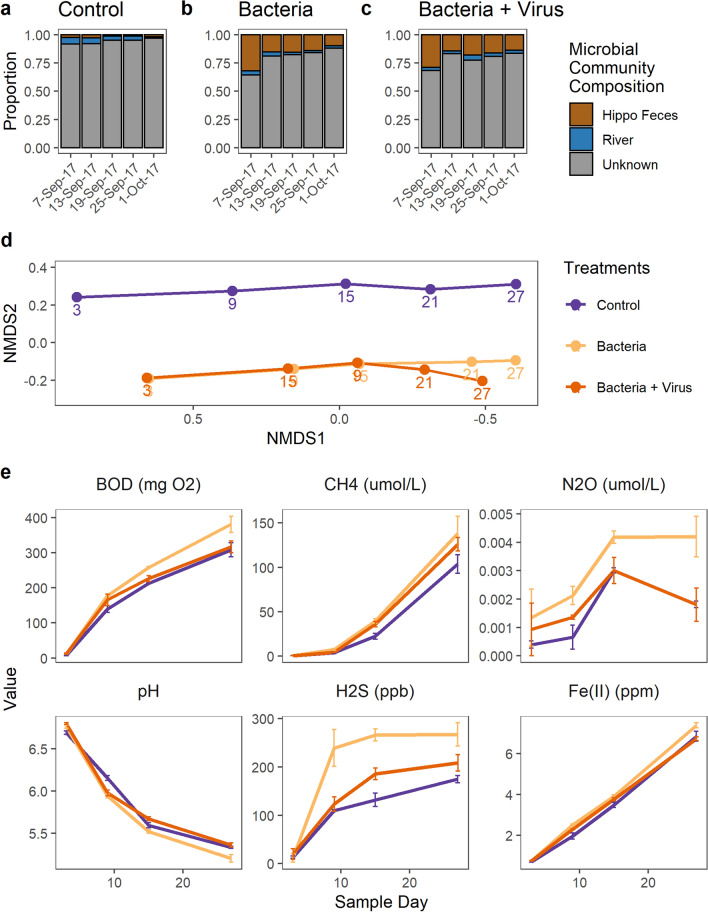
Active aquatic microbial community composition and biogeochemistry from the experimental treatments. The proportion of the active microbial community from hippo feces, upstream river water, or of unknown origin for **(a)** Control, **(b)** Hippo feces bacteria, no viruses and **(c)** Hippo feces bacteria + virus treatments. **(d)** NMDS of the Bray Curtis Dissimilatory matrix of the active aquatic microbial communities from the three experimental treatments across time. Numbers 1–27 represent the five sampling days for the experiment. NMDS Stress 0.09. **(e)** Biogeochemical variables reflecting microbial catabolism, showing mean concentrations (N = 3) and standard deviations as error bars (additional variables are provided in Supplementary Fig. S12).

We found distinct differences in the composition of the active aquatic microbial communities through time relative to the control (Fig. 5a–d). The proportion of microbes from hippo feces was largest on the first day after the start of the experiment (Day 3, September 7th, 2017) and declined thereafter. The total taxa (including active and inactive microorganisms) included more taxa derived from upstream than we detected in just the active taxa (Fig. 5a–c, Supplementary Fig. S11). We found a statistically significant effect of treatment on biogeochemical variables including Fe(II), pH, H_2_S, BOD, CH_4_, and SO_4_^2−^ (Table 1). The bacteria treatment had higher concentrations of Fe(II) and lower pH compared to the bacteria + virus treatment. The bacteria treatment also had higher concentrations of BOD, H_2_S, Fe(II) and lower pH in compared to the control. There were no differences in biogeochemistry between the control and the bacteria + virus treatment.

**Table 1:** Linear mixed effect model results for each biogeochemical variable.

Biogeochemical variable	LME—treatment effect		Pairwise posthoc test treatment		
	F-value	p-value	Control	Bacteria	Bacteria + virus
Fe(II)	24.200	**0.001**	**a**	**b**	**a**
pH	15.999	**0.004**	**a**	**b**	**a**
H_2_S	11.104	**0.010**	**a**	**b**	**ab**
BOD5	9.557	**0.014**	**a**	**b**	**ab**
CH_4_	6.177	**0.035**	a	a	a
SO_4_^2−^	5.902	**0.038**	a	a	a
Chl *a*	4.827	0.056	a	a	a
Cl^−^	4.081	0.076	a	a	a
SRP	2.408	0.171	a	a	a
NO_3_^−^-N	1.406	0.316	a	a	a
F^−^	1.339	0.330	a	a	a
N_2_O	1.232	0.356	a	a	a
NH_4_^+^-N	1.225	0.358	a	a	a
K^+^	0.964	0.434	a	a	a
Na^+^	0.946	0.440	a	a	a
Br^−^	0.855	0.471	a	a	a
Ca^2+^	0.673	0.545	a	a	a
DOC	0.272	0.771	a	a	a
Mg^2+^	0.247	0.789	a	a	a
ORP	0.233	0.799	a	a	a
CO_2_	0.233	0.799	a	a	a

Variables are ordered from top to bottom by statistically significant differences. Letters indicate significant differences determined by the pairwise posthoc test using Tukey’s adjusted p-value for multiple comparisons.

Significant values are in [bold].

We found that the microbial communities in the bacteria and the bacteria + virus treatments were relatively similar across time until the last two sample days (sample day 23 and 27) when they began to diverge (Bray–Curtis dissimilatory matrix and NMDS ordination; Fig. 5d). Microbial communities from the last time point in the experiment consisted primarily of obligate anaerobes rather than the more diverse communities associated with the hippo gut microbiome.

## Discussion

Hippos excrete microbes into the water of hippo pools, and a portion of the microbial taxa continues to function and may appreciably alter environmental biogeochemical processes (Fig. 1)^23^. The collective hippo-environmental microbiome, the *meta-gut*, is reinforced through the constant loading of hippo feces into hippo pools, which depletes dissolved oxygen and increases the similarity between the host gut and the aquatic environment.

Biogeochemistry within pools was driven by interactions between hippo loading and environmental characteristics of the pool. Microbial communities in high-subsidy hippo pools were strongly associated with higher levels of soluble reactive phosphorus (Fig. 2b), which is likely due to inputs of P in hippo urine and to the release of SRP from sediments under anoxic conditions. The oxic waters of low-subsidy hippo pools had higher concentrations of nitrate than found in the main river, and there was higher sulfate found in the medium-subsidy pools in the tributaries^23^. The tributaries are likely draining catchments that have more geological sources of sulfur, while the higher nitrate concentrations in the low-subsidy hippo pools in the river may be due in part to oxic conditions that result in less denitrification. These biogeochemical differences likely interact with the rate of loading by hippos to alter microbial communities within the pools, which in turn may further alter the biogeochemistry.

We hypothesized that hippos within a pool would have more similar gut microbiomes than hippos across pools. Data from this study did not support that hypothesis—hippos from the same high-subsidy pool did not have more similar microbiomes to one another than to hippos from other high-subsidy pools (Supplementary Fig. S3). However, our sample size was small (N = 10, 3 feces samples per 3 pools and one additional sample near another pool), and samples were only collected near high-subsidy pools soon after a flushing flow. It is possible that similarity amongst individual gut microbiomes is more likely to develop over time between flushing flows or during dry periods when hippos congregate in larger numbers within the remaining pools.

The proportion of the active microbial community from the hippo gut in high-subsidy hippo pools increased over time between flushing flows. At most time points, the predominant source for the active microbial communities was unknown (Fig. 4a–c). Given that high-subsidy hippo pools stay anoxic for prolonged periods of time, the unknown taxa are likely free-living anaerobes that have grown in the anoxic environment in the bottom of the hippo pools and cannot be ascribed specifically to originating either from the upstream environment or the hippo gut. As hippo loading continues after a flushing flow, the hippo gut microbial community likely outcompetes certain free-living anaerobes resident in the pool while coalescing into a new community derived in part from the hippo gut microbiome. We found distinct taxa derived from the hippo gut microbiome in the bottom of hippo pools (Supplementary Fig. S8). The high-subsidy pool with the highest proportion of hippo gut microbiome derived communities (PRHP) also had the highest concentrations of indicators of microbial catabolism, including BOD, NH_3_ + NH_4_^+^, SRP, DOC, CH_4_, and CO_2_ compared to the other high-subsidy pools^23^. *Macellibacteroides* and *Odoribacter* were shown to be actively functioning in the bottom waters of PRHP and increasing in relative abundance between flushing flows (Supplementary Fig. S8). *Macellibacteroides* was also found to be strongly correlated with higher BOD, H_2_S, Fe(II), DOC, CO_2_, CH_4_, K^+^, SRP, TN, TP, NH_4_^+^-N, conductivity, Mg^2+^, and Ca^2+^ (Supplementary Fig. S7). The hippo gut microbial communities either facilitate a more reducing environment ex situ and/or thrive better within it.

Our mesocosm experiment showed that a higher proportion of active hippo gut bacteria was associated with higher concentrations of BOD, CH_4_, N_2_O, and H_2_S, suggesting that hippo gut microbes are driving some of biogeochemical differences we observed in high-subsidy hippo pools. In our experiment, the proportion of the microbial community derived from the hippo gut microbiome decreased over time, in contrast to field measurements showing an increase in gut microbes over time between flushing flows (Figs. 4, 5). This difference may be because continual inputs of hippo feces and gut microbes, which are relatively constant in high-subsidy hippo pools^29^, may be necessary to outcompete other communities. It may also be that input of hippo gut microbes directly into an anoxic environment, as occurs in high-subsidy hippo pools in the field, may increase successful colonization of the pool environment. During the experiment, we loaded the hippo gut microbiome into an oxic environment at the beginning of the experiment and the bottles received no further loading. The anoxic conditions frequently present in high-subsidy hippo pools may be necessary to increase the survival of hippo gut microbes^16^. The final microbial communities in all treatments were composed primarily of taxa not derived from the hippo gut, such as *WCHB1-32* (10% average across treatments) and *Holophagaceae* (6%).

Microbially-mediated transformations of biologically important elements were altered in the presence of bacteriophages from the hippo gut. The experimental treatments with a low viral load (bacteria treatment) had higher dissolved Fe(II) and lower pH than the treatments with a high viral load (bacteria + virus treatment) (Fig. 5e). We also found higher concentrations of BOD and H_2_S in the bacteria treatment than the control, with no difference between the bacteria + virus treatment and control. We hypothesize that the effect of bacteriophages could be greater when the transmission of feces occurs directly into an anoxic environment, as these conditions would decrease the activity of protists that could consume the bacteriophages^30,31^. In both the hippo gut and in hippo pools, bacteriophages may exert particularly important control over the microbial community due to the lack of protists in anoxic environments and the likely high viral load of hippo feces^30–32^. Viral lysis of bacteria has been shown to alter biogeochemical processes such as nitrogen remineralization^33^. Our experimental results suggest that bacteriophages may be inhibiting the activity of bacteria in the hippo pools, including those directly relevant to biogeochemical cycling.

Hippos subsidize tropical rivers through carbon and nutrient loading^20,21,23,24,34,35^, and here we show that they also subsidize it through loading of their gut microbiome, which uniquely ties hippos to the health of the entire riverine ecosystem. Gut microbiota provide a number of important functions that hosts are either unable to do or can do only in a limited capacity, including synthesizing amino acids, fatty acids, vitamins, and the metabolism of carbohydrates^36^. When hippos defecate into pools, many of these by-products are made directly available to aquatic life, which may utilize them as a subsidy^37^. Aquatic insects and fishes near hippo pools also may participate in the *meta-gut* system through the colonization of hippo gut-derived microbes into their guts. Fish gut microbiomes typically vary in response to environment and diet^38^, and multiple species of fish in tropical rivers consume hippo feces^35^. If hippo gut-derived microbes are able to establish within the fish gut, they may aid in the digestion of hippo feces, or be transported to new locations by fish movements. Intriguingly, a previous meta-analysis of fish microbiomes found an unexpected similarity in fermentative bacteria between fish gut communities and vertebrate gut communities, highlighting the potential for this effect^39^. Aquatic insects also have been known either to ingest gut microbiota from the environment that can then aid directly in digestion or to rely on external microbial decomposition to process recalcitrant organic matter prior to ingestion^40^.

Hippos provide an ideal case study for examining the *meta-gut* system in wild populations, as they congregate in high densities in pools that quickly become anoxic due to a subsidy overload^23,25^. Transmission of the microbiome to the external environment may be strongest when transmission occurs directly between similar environments, such as from the gut of an animal to an anoxic aquatic environment that protects the microbiome from external stressors (atmospheric oxygen, ultraviolet radiation, adverse temperatures, etc.)^41^. This dynamic may occur broadly in other animals that wallow^42–44^, share latrine sites^45^, or share a common aquatic environment that can experience hypoxia/anoxia such as mussels^46,47^. Extinct lineages of hippo-like animals or freshwater megafauna with similar environmental niches may have produced similar dynamics prior to their extinctions^48–50^, suggesting that species losses of current freshwater megafauna, many of which are threatened with extinction or locally extirpated, could have even broader impacts on ecosystem functioning than previously anticipated^50–52^.

## Conclusions

Altogether our research demonstrates that community coalescence between the hippo gut microbiome and the river microbiome can occur, forming a *meta-gut* system in which the hippo gut microbiota can continue to function ex situ in certain environmental contexts. Thus, the role of hippos in subsidizing tropical rivers extends beyond the loading of nutrients and organic matter to also include the successful transference of microbial communities that may influence biogeochemical cycling. In hippo pools, two factors appear necessary for the *meta-gut* system to develop. First, there is a continual loading of waste products (organic matter and nutrients) and microbial communities. Without continual loading, the resulting microbial community may coalesce into an anaerobic community that does not represent the functioning gut microbial communities from the donor host. Second, loading occurs in an environmental patch that has similar environmental characteristics to the gut, which in this case develops in response to organic matter loading by hippos. Our research demonstrates that some animals can function as mobile anaerobic bioreactors, consuming organic matter, accumulating and selecting for microbes in their guts, and moving and depositing waste products and gut microbes across the landscape, where the microbes may affect ecosystem functioning of the external environment.

## Methods

### Microbial community sampling

#### Hippo gut microbiome

We characterized the microbial communities in the hippo gut by collecting ten samples of fresh hippo feces adjacent to four hippo pools early in the morning (prior to desiccation by the sun) in September 2017. We collected feces from different pools and locations adjacent to the pool to include the feces of different individuals so we could estimate the similarity of the gut microbiome among individuals and across the landscape. The four hippo pools are sufficiently far apart that there was likely no intermixing of hippos among them.

Each individual hippo feces sample was gently homogenized by hand and then the liquid was gently squeezed from the coarse particulate organic matter. A portion of the liquid (approximately 10 mL) was vacuum filtered through a Supor polysulfone membrane (0.2-µm pore size; Pall, Port Washington, NY, USA). After approximately 10 mL had filtered through and the filter was dry, 15 mL of RNALater was gently poured onto the filter and allowed to contact the collected biomass on the filter for 15 min before being removed by filtration. The filter was stored dry in a sterile petri dish and transferred to a refrigerator within several hours, then to a − 20 °C freezer for storage within several days.

During the July 2016 survey of hippo pools, we collected an additional two samples of fresh hippo feces near a high-subsidy hippo pool and filtered approximately 10 mL of the liquid portion after homogenization as detailed above. The filter was then folded twice to preserve the biomass on the filter and stored in 14 mL of RNALater.

#### Aquatic ecosystem

We characterized the microbial communities in the water column of hippo pools across a gradient of hippo subsidy (July 2016, N = 12 pools). We collected samples from the upstream, downstream, surface, and bottom of both pools containing hippos and pools that lacked hippos. Subsamples were also analyzed for biogeochemical variables (details provided below). We also collected water samples in four of the high-subsidy hippo pools every 2–3 days starting immediately after a flushing event until the next flushing event (August and September 2017, Supplementary Fig. S1)^23^. The number of hippos, discharge and volume for each pool are presented in Dutton et al (2020)^23^.

We sampled the aquatic microbial community and biogeochemical variables along a longitudinal transect down both the Mara and Talek rivers (Supplementary Fig. S1, Supplementary Table S2). For the Mara River, we sampled an approximately 100-km transect along a gradient of hippo numbers (N = 10 locations, from 0 to ~ 4000 hippos). For the Talek River, we sampled an approximately 30-km transect to the confluence with the Mara (N = 8 locations, from 0 to 700 hippos). Mara River sites 9 and 10 are downstream of the confluence with the Talek River. Water samples were collected from each site in a well-mixed flowing section away from any hippo pools.

Aquatic microbial samples were collected by filtering water samples through a Supor polysulfone filter (0.2-µm pore size; Pall, Port Washington, NY, USA) and then preserving the filter in RNALater Stabilization Solution (Ambion, Inc., Austin, TX, USA). In 2017, the filters were preserved with RNA Later and then frozen for analysis.

### Mesocosm experiment

We collected river water from the Mara River upstream of the distribution of hippos and placed it in 45 1-L bottles in a large water basin covered by a dark tarp to help regulate temperature and prevent algal production. Bottles were randomly assigned to the control, bacteria, and bacteria + virus treatments. We collected fresh hippo feces from multiple locations adjacent to the Mara River. After homogenization, half of the hippo feces was sterilized in a pressure cooker, which testing confirmed had similar sterilization results as an autoclave^53^ (see Supplementary Materials). Five grams of sterilized hippo feces was placed into each bottle to provide an organic matter substrate without viable bacteria or viruses. The unsterilized hippo feces was expressed, and the resulting liquid was filtered through 0.7-µm GF/F filters (0.7-µm pore size; Whatman, GE Healthcare Life Sciences, Pittsburgh, PA, USA) and 0.2-µm Supor filters to physically separate the bacteria (on the filter papers) from the viruses (in the filtrate). Half the filtrate was then sterilized with a UV light treatment (Supplementary Fig. S4). The UV light treatment did not significantly alter DOC quality (see Supplementary Materials).

We prepared 15 bottles for each of three treatments—control, bacteria, and bacteria + virus—as follows: *Control* Unfiltered river water, 5 g wet weight sterilized hippo feces, and two blank Supor filters; *Bacteria* Unfiltered river water, 5 g wet weight sterilized hippo feces, two Supor filters containing bacteria, and 4 mL sterilized filtrate; *Virus* Unfiltered river water, 5 g wet weight sterilized hippo feces, two Supor filters containing bacteria, and 4 mL unsterilized filtrate containing viruses.

We conducted the experiment for 27 days from September to October 2017. We terminated the experiment after 27 days because we were trying to replicate the microbial communities in hippo pools as best as we could and the hippo pools rarely go more than 1 month before they are flushed out by a flood^25^. Initial microbial samples of the river water, hippo feces bacteria and hippo fecal liquid filtrate were taken on day 0, and three replicate samples per treatment were destructively sampled on day 3, 9, 15, 21, and 27. During each time step, the microbial communities were sampled using the methods detailed above, and chemical analyses were done on the water samples as described below. We also measured chlorophyll a, dissolved oxygen, temperature, conductivity, total dissolved solids, turbidity, and pH with a Manta2 water quality sonde (Eureka Water Probes, Austin, TX, USA).

### Microbial community characterization

We used 16S rRNA sequencing to characterize the active microbial communities. We extracted both DNA and RNA from our preserved samples, then used RNA to synthesize cDNA to represent the “active” microbial community and the total DNA in the sample to represent the “total” microbes present, including those that may not be actively replicating^54^. Due to the continual loading of hippo feces into pools and the long half-life of DNA, we would expect there to be significant quantities of microbial DNA derived from hippo feces within the pools. However, there would be less accumulation of RNA because of RNA’s shorter half-life. The active communities identified through this RNA-based approach are the ones that would potentially contribute to ecosystem function^55^ as indicated by the protein synthesis potential, although relationships between activity and rRNA concentrations in individual taxa within mixed communities can vary^56^. Nevertheless, this method provides an overall characterization of the microbial community’s potential activity.

We used the Qiagen RNeasy Powerwater Kit (Qiagen, Hilden, Germany) to extract the DNA and RNA from the material on the filter using a slightly modified manufacturer’s protocol to allow for the extraction of both DNA and RNA. After extraction, we split the total extracted volume (100 µL per sample) into two groups. We treated one group with the DNase Max Kit (Qiagen, Hilden, Germany) to remove all DNA and serve as the RNA group of samples.

We used the RNA group of samples to synthesize cDNA using the SuperScript III First Strand Synthesis Kit (Invitrogen, Carlsbad, CA, USA). DNA and cDNA were quantified using the PicoGreen dsDNA Assay Kit (Molecular Probes, Eugene, OR, USA) then normalized to 5 ng/µL. Amplicon library preparation was done using a dual-index paired-end approach^57^. We amplified the V4 region of the 16S rRNA gene using dual-index primers (F515/R805) and AccuPrime Pfx SuperMix (Invitrogen, Carlsbad, CA, USA) in duplicate for each sample using the manufacturer’s recommended thermocycling routine.

Samples were then pooled, purified and normalized using the SequelPrep Normalization Plate Kit (Invitrogen, Carlsbad, CA, USA). Barcoded amplicon libraries were then sequenced at the Yale Center for Genome Analysis (New Haven, CT, USA) using an Illumina Miseq v2 reagent kit (Illumina, San Diego, CA, USA) to generate 2 × 250 base pair paired-end reads.

Sampling took place in 2016 and 2017 and involved two separate sequencing runs. The first sequencing run included negative controls and a mock community (D6306, Zymo Research, Irvin, CA, USA). The second sequencing run included negative controls, a mock community (D6306), and a single *E. coli* strain. In both runs, the mock community and single *E. coli* strain were well reconstructed from the sequences, and there was minimal contamination in the negative controls, mock community and *E. coli* strain.

From those two sequencing campaigns, we received over 2 million raw sequences from the first campaign and over 7 million raw sequences for the second campaign. For the microbial community analyses, only samples collected and sequenced during the same campaign are analyzed together to prevent preservation or sequencing biases. However, samples within the two separate campaigns were preserved and sequenced using identical methods with only a minor modification (mentioned above) to increase the preservation of genetic material.

We de-multiplexed sequenced reads then removed barcodes, indexes, and primers using QIIME2^58^. We used DADA2 with a standard workflow in R^59^ to infer exact sequence variants (ESV) for each sample^60^. We assigned taxonomy using a naïve Bayesian classifier and the SILVA training set v. 128 database^61,62^. We removed potential contamination in samples from both campaigns by using the statistical technique in the R package, d*econtam*^63^. We used Phyloseq to characterize, ordinate, and compare microbial communities^64^ with their standard workflow^59^.

### Chemical analyses

All water samples collected in the field and in the experiment were analyzed for dissolved ferrous iron (Fe(II)), hydrogen sulfide (H_2_S), dissolved organic carbon (DOC), inorganic nutrients, major ions, dissolved gases, and biochemical oxygen demand following the standard methods provided in detail in Dutton et al (2020)^23^.

### Statistical analyses

We computed all statistical analyses in the R 4.1.1 statistical language in RStudio 2021.09.0 using α = 0.05 to determine significance^65,66^. Error bars in the figures represent standard deviation of the means. All data and R code for the statistics and data treatments are provided in the Mendeley Data Online Repository^67^.

We used the Bray–Curtis dissimilarity matrix followed by ordination with NMDS to examine differences between individual hippo gut microbiomes; between low-, medium-, and high-subsidy hippo pools; and between a gradient of hippo pools and the environment. We used a CCA to test for the influence of biogeochemical drivers on microbial community composition using biogeochemical data that were previously published but collected concurrently with this study^23^. We constrained the CCA ordination by soluble reactive phosphorus, nitrate, methane, BOD, and sulfate, which were all previously shown to be important drivers in the variation between pools^23^. We used PERMANOVA and PERMDISP to test for significant differences between groups^68^.

We compared aquatic microbial communities from the bottom of high-subsidy hippo pools (N = 15), from hippo feces (N = 10, the hippo gut microbiome) and upstream of high-subsidy hippo pools (N = 15, free of hippo gut microbiome influence) using the Bray–Curtis dissimilarity matrix on the relative abundances for the active aquatic microbial communities collected from the different sample types followed by ordination with NMDS. 95% confidence ellipses were generated. We then determined the active taxa that were shared between the hippo gut microbiome (hippo feces) and the bottom of the high-subsidy hippo pools and not present in the upstream samples from high-subsidy hippo pools.

We used LEfSe to calculate the differential abundance of microbial taxa between upstream (N = 14), downstream (N = 16), at the surface (N = 17) and at the bottom (N = 14) of hippo pools and calculated their effect size^69^. We then calculated the correlation of microbial taxa to the measured biogeochemistry using Pearson’s correlation coefficient with a false discovery rate corrected p-value in the microeco R package^70^.

We used SourceTracker to quantify the contribution of the hippo gut, upstream waters, or unknown sources to the active aquatic microbial communities in the bottom waters of three of the high-subsidy hippo pools between flushing flows^71^. We also used the Bray–Curtis dissimilarity matrix followed by ordination with NMDS to examine changes in the active aquatic microbial communities in one of the high subsidy hippo pools through time after flushing flows.

For the experiment, we calculated the Bray–Curtis dissimilatory matrix followed by ordination with NMDS for the active aquatic microbial communities over time in each of the three experimental treatments. We used SourceTracker to determine the proportion of the active aquatic microbial community in each treatment that originated from the hippo gut, the river water, or unknown sources^71^. We analyzed the biogeochemical differences among experimental treatments by fitting a linear mixed effects model for each of the biogeochemical variables throughout the experiment with the nlme package in R^72^. We fit the model with the restricted maximum likelihood method and a continuous autoregressive temporal correlation structure with sample day as the repeated factor. Treatment and time were fixed effects and individual bottles were treated as random effects. We conducted a pairwise post-hoc test with an ANOVA and the emmeans package in R to estimate marginal means with a Tukey adjusted p-value for multiple comparisons^73,74^.

## Supplementary Information

Supplementary Information.

## Data Availability

All data and R code for the statistics and data treatments are provided in the Mendeley Data Online Repository^67^.
