# Supplementary material for: The meta-gut: community coalescence of animal gut and environmental microbiomes
Source: Sci Rep. 2021 Nov 30;11:23117. doi: 10.1038/s41598-021-02349-1 (PMC8633035; doi:10.1038/s41598-021-02349-1)
Supplement: Supplementary file 1 — Supplementary Information. [file 41598_2021_2349_MOESM1_ESM.pdf]

Supplementary Information for

The meta-gut: community coalescence of animal gut and environmental microbiomes

Christopher L. Dutton, Amanda L. Subalusky, Alvaro Sanchez, Sylvie Estrela, Nanxi Lu, Stephen K. Hamilton, Laban Njoroge, Emma J. Rosi, David M. Post

Corresponding Author: Christopher L. Dutton

Email: clddutton@gmail.com

This PDF file includes:

Figures S1 to S13

Table S1 to S2

Supplementary Text

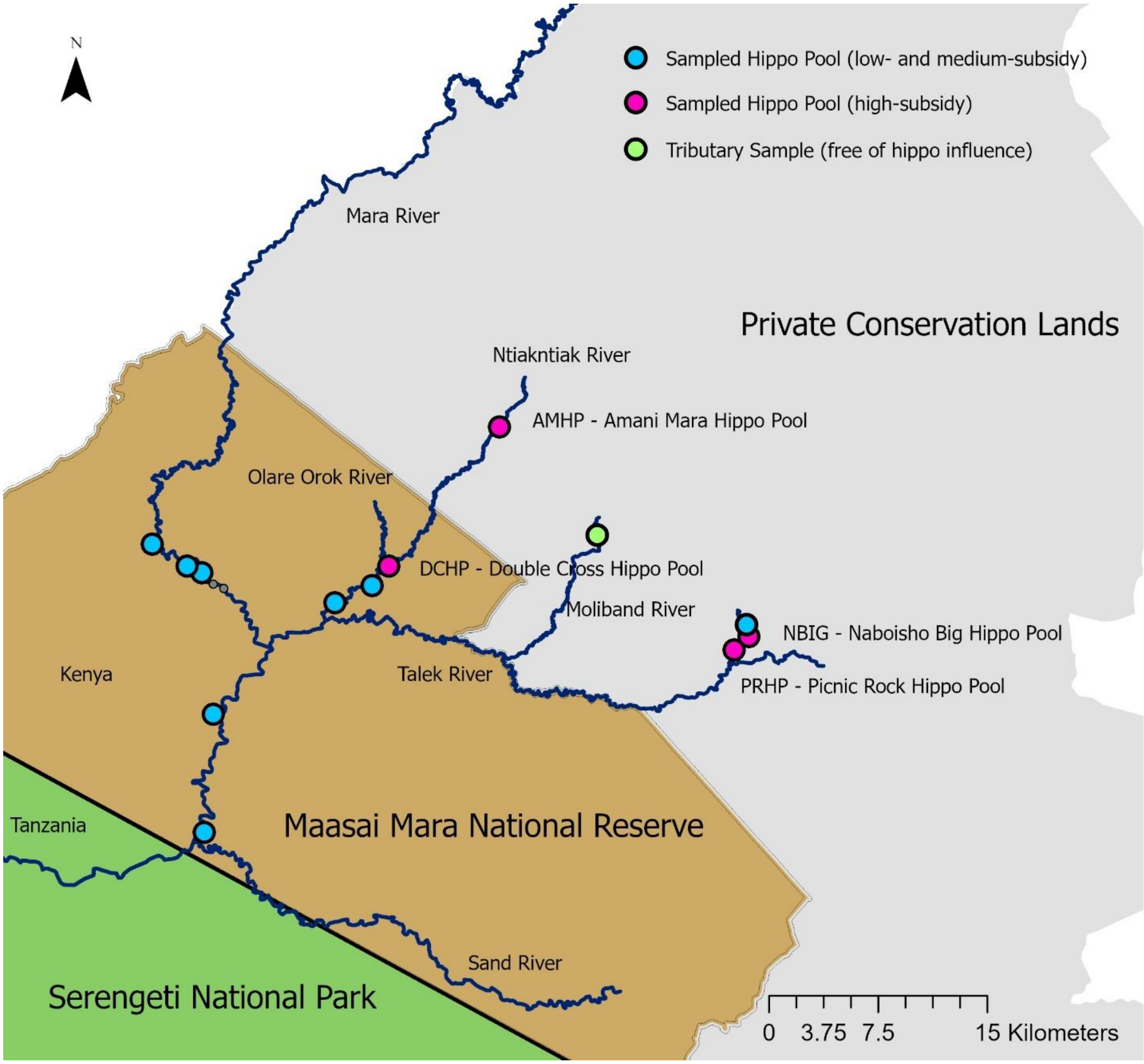

Figure S1: Hippo pools sampled across a gradient of hippo subsidy (blue and pink circles) and four high-subsidy hippo pool sampled during transitions between aerobic and anaerobic states (pink circles). One sample was collected from a tributary that was free of hippo influence (green circle). This map was generated using ESRI ArcMap 10.4.1 (https://www.esri.com/en-us/home).

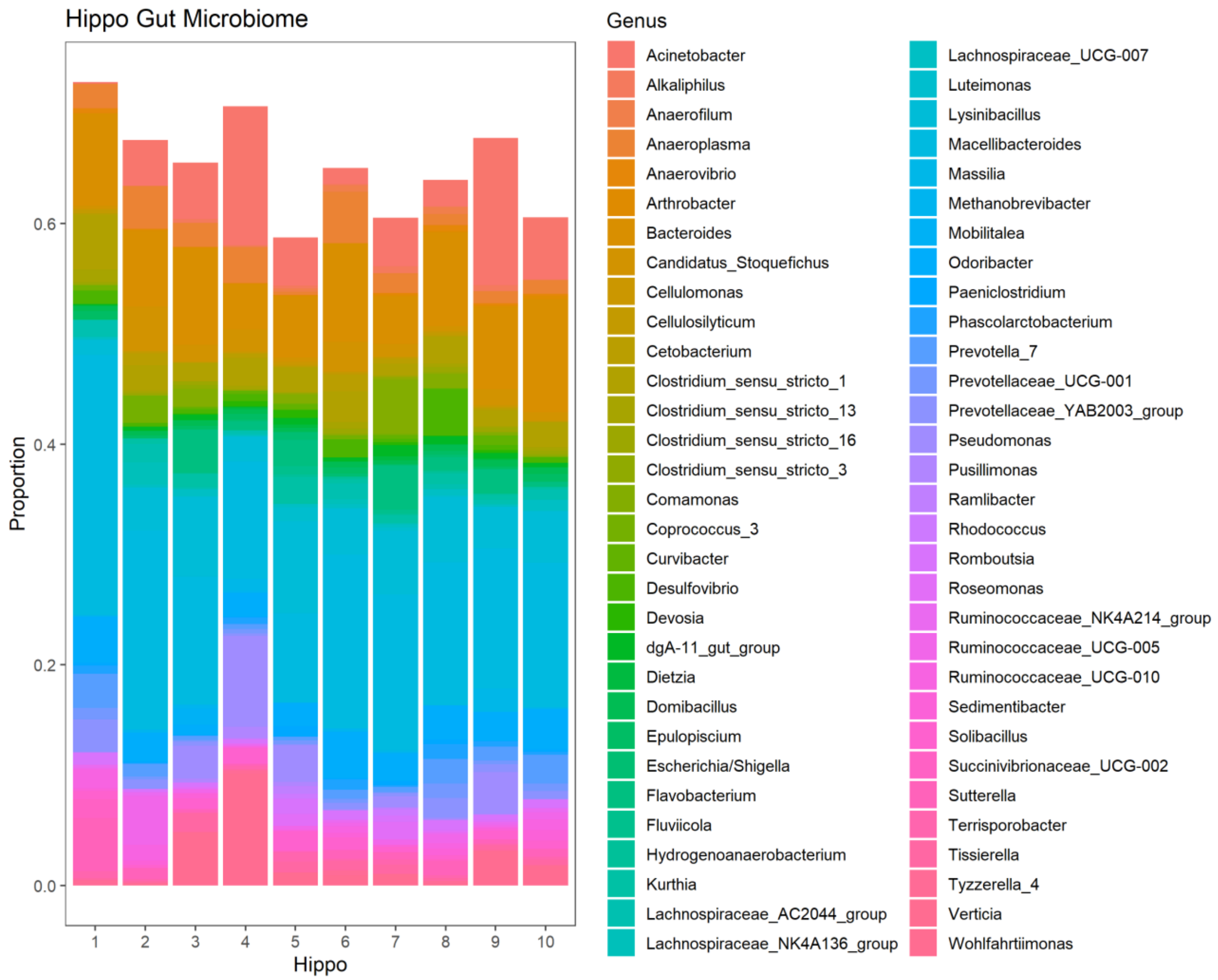

Figure S2: Genus proportions of the active microbial communities present in the guts of 10 individual hippos.

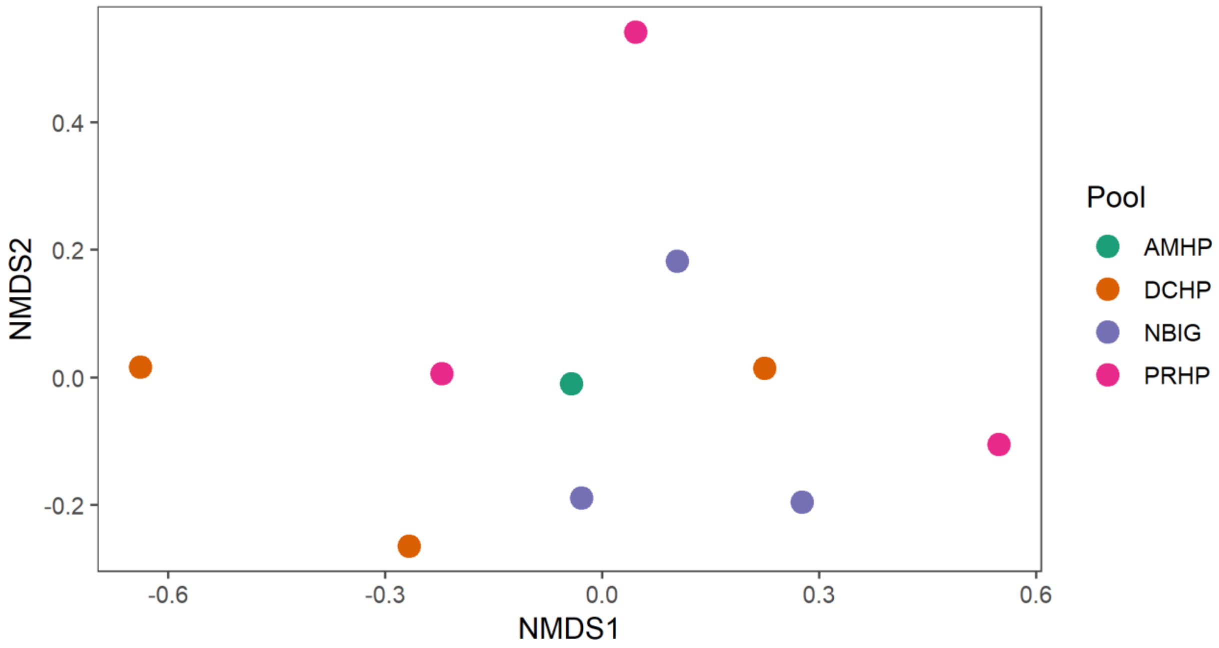

Figure S3: NMDS ordination the Bray-Curtis dissimilarity matrix of the active microbial community in hippo feces (N=10) collected near four high-density hippo pools (AMHP N=1, DCHP N=3, NBIG N=3, PRHP N=3).

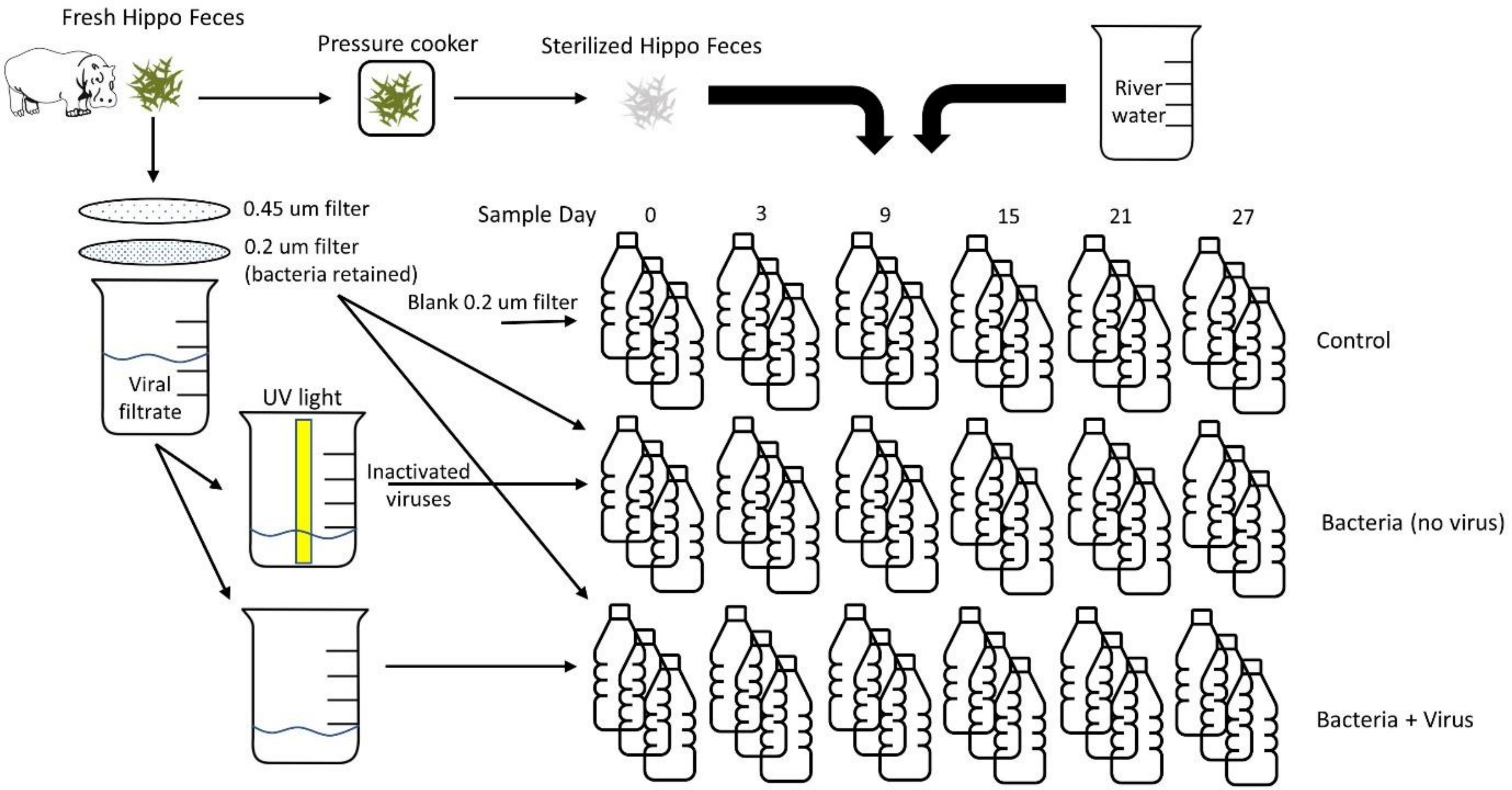

Figure S4: Microcosm experimental design to characterize the changing microbial communities as a hippo pool goes anoxic, to elucidate the role of microbial taxa associated with the hippo gut in driving biogeochemical changes, and to examine the impact of fecal bacteriophages from the hippo gut on bacterial communities and biogeochemical processes.

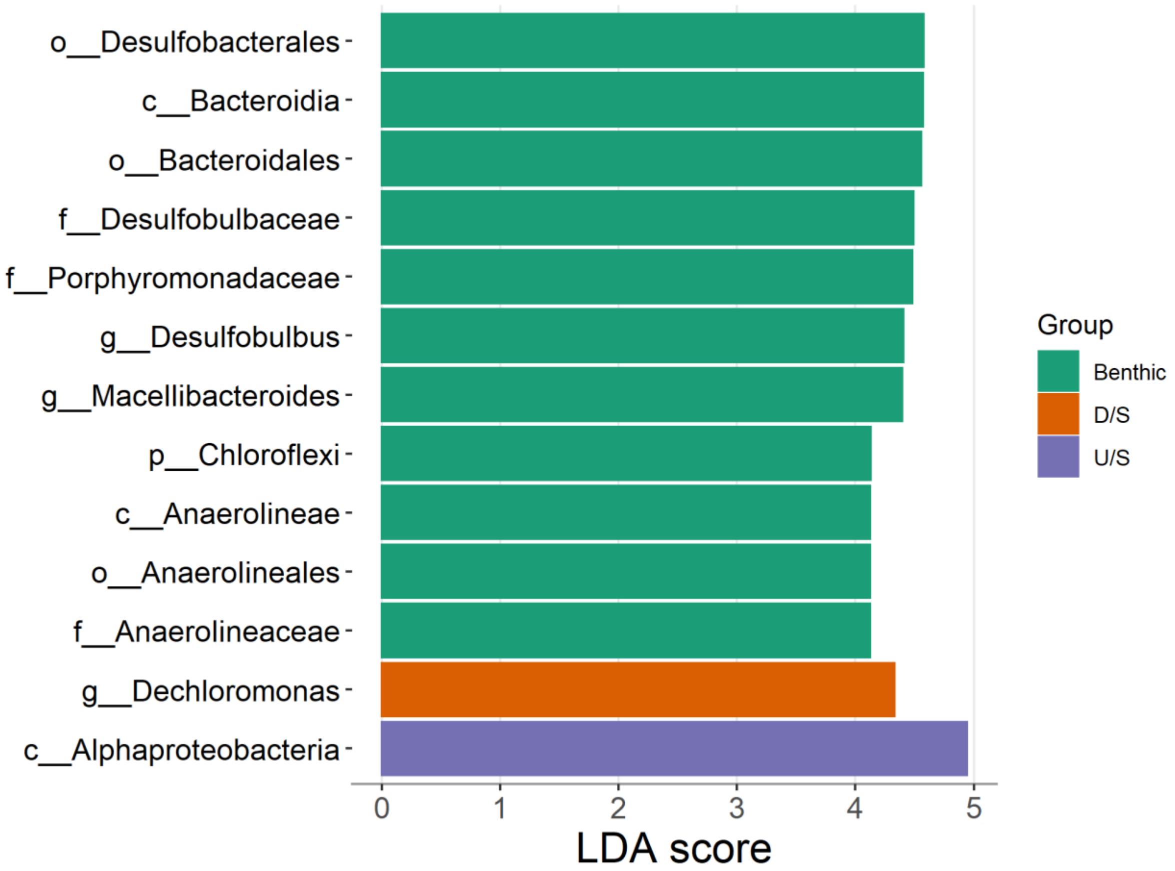

Figure S5: Linear discriminant analysis score from LEfSe. These taxa have a differential abundance between upstream, downstream, surface and bottom of hippo pools.

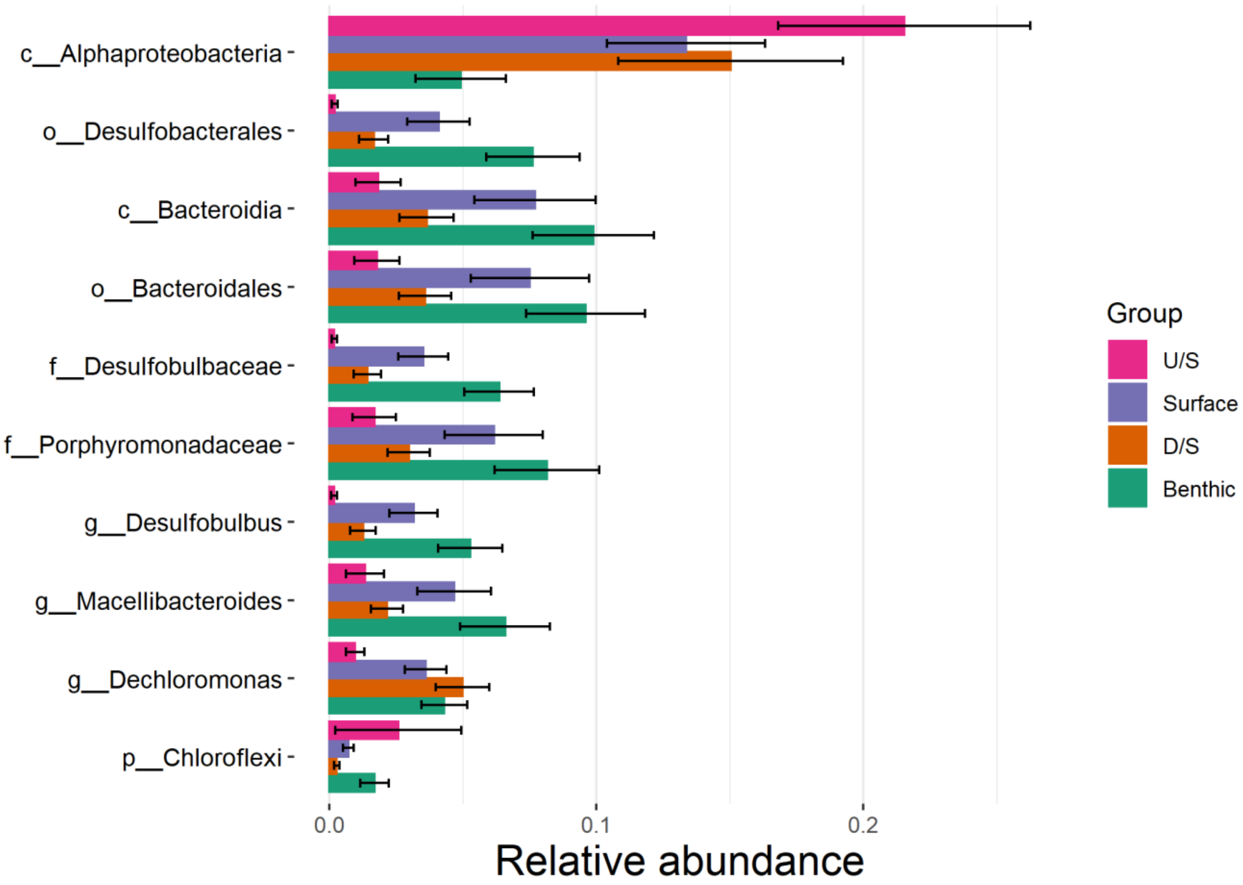

Figure S6: Relative abundance between the four locations at a hippo pool for the 10 groups of microbial taxa that have differential abundance (see Supplementary Fig. S5).

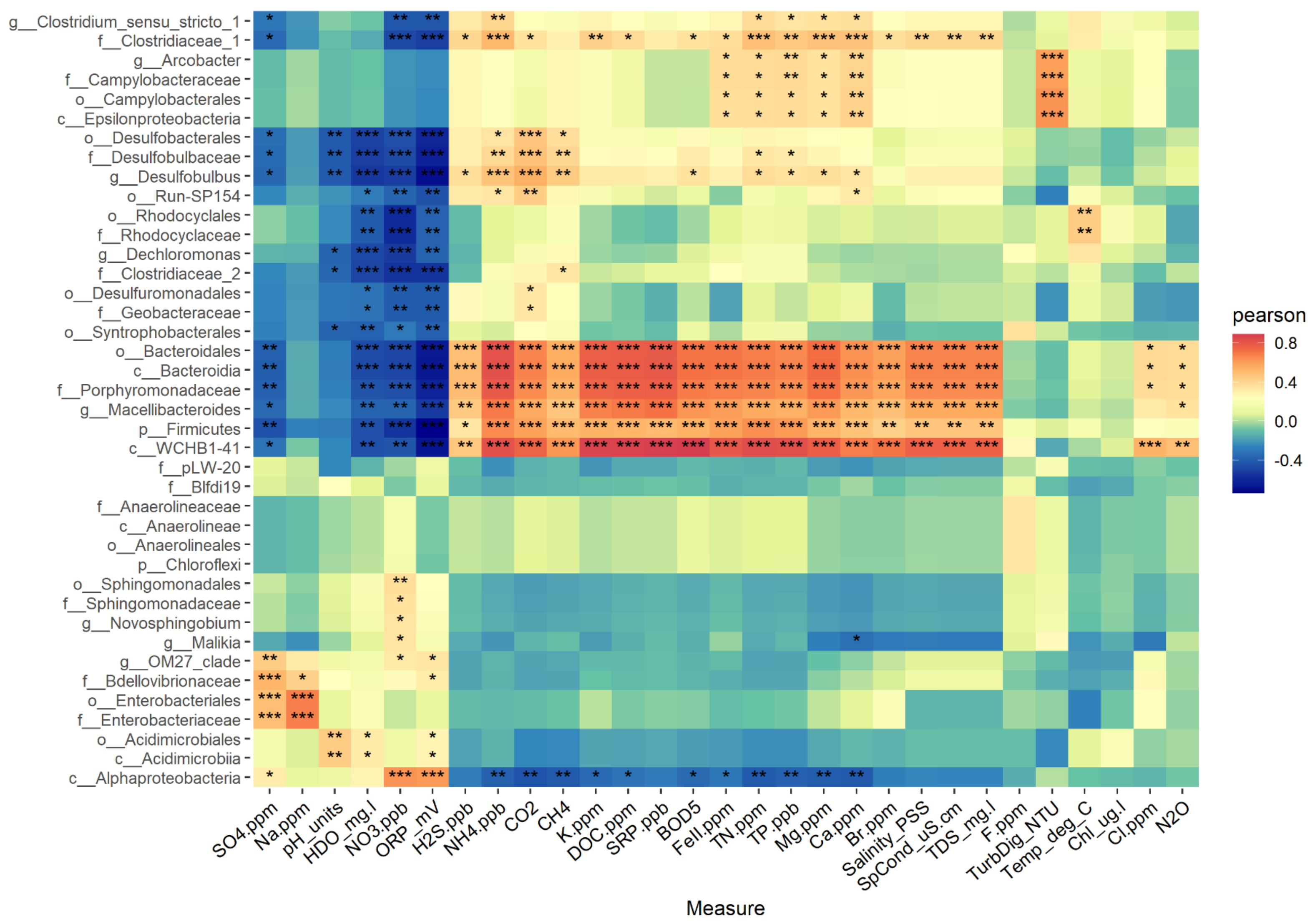

Figure S7: Pearson correlation heatmap between indicator taxa and measured biogeochemistry within and around hippo pools. * :P < 0.05,∗∗: P < 0.01;∗∗∗: P < 0.001.

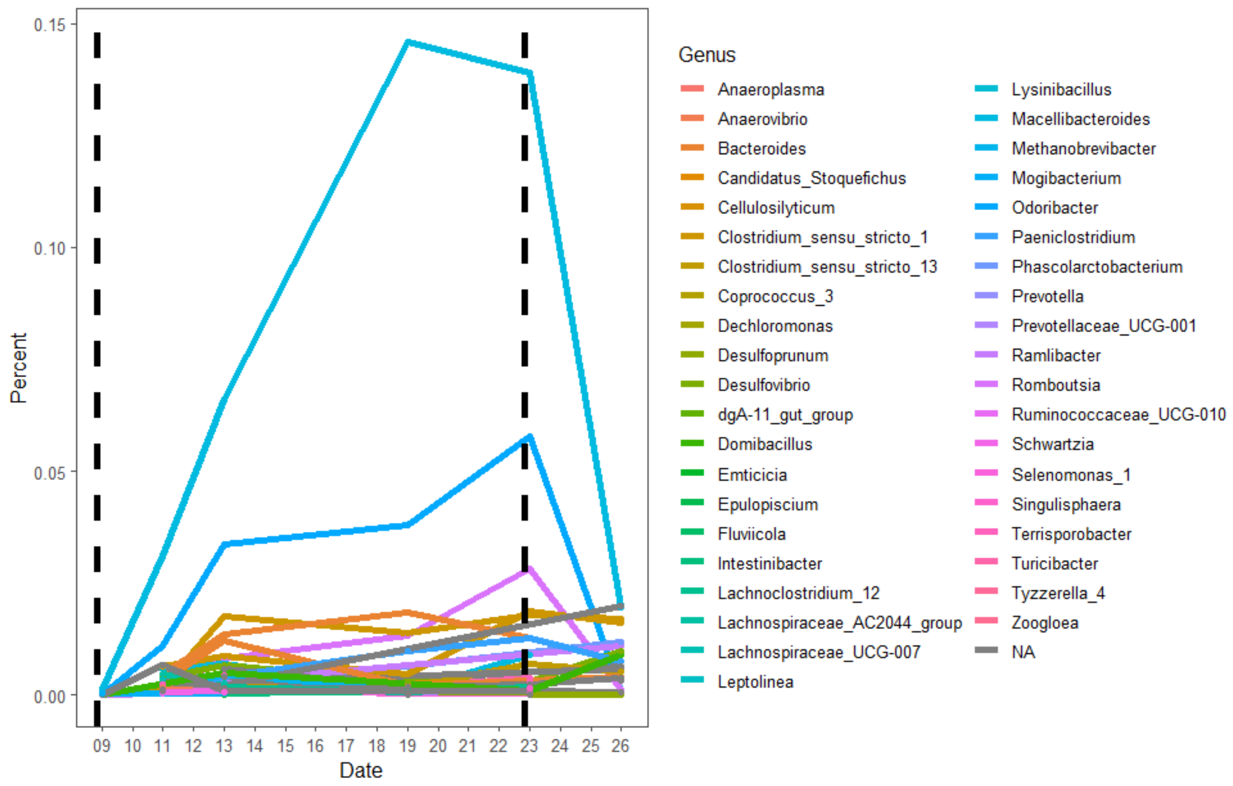

Figure S8: The active microbial taxa (genus) from the bottom of the hippo pool as attributed to hippo feces by SourceTracker from PRHP during the flushing flows. Dashed lines indicate the flushing flows. The first flushing flow occurred on 8-Aug-17. The second flushing flow occurred on 25-Aug-17.

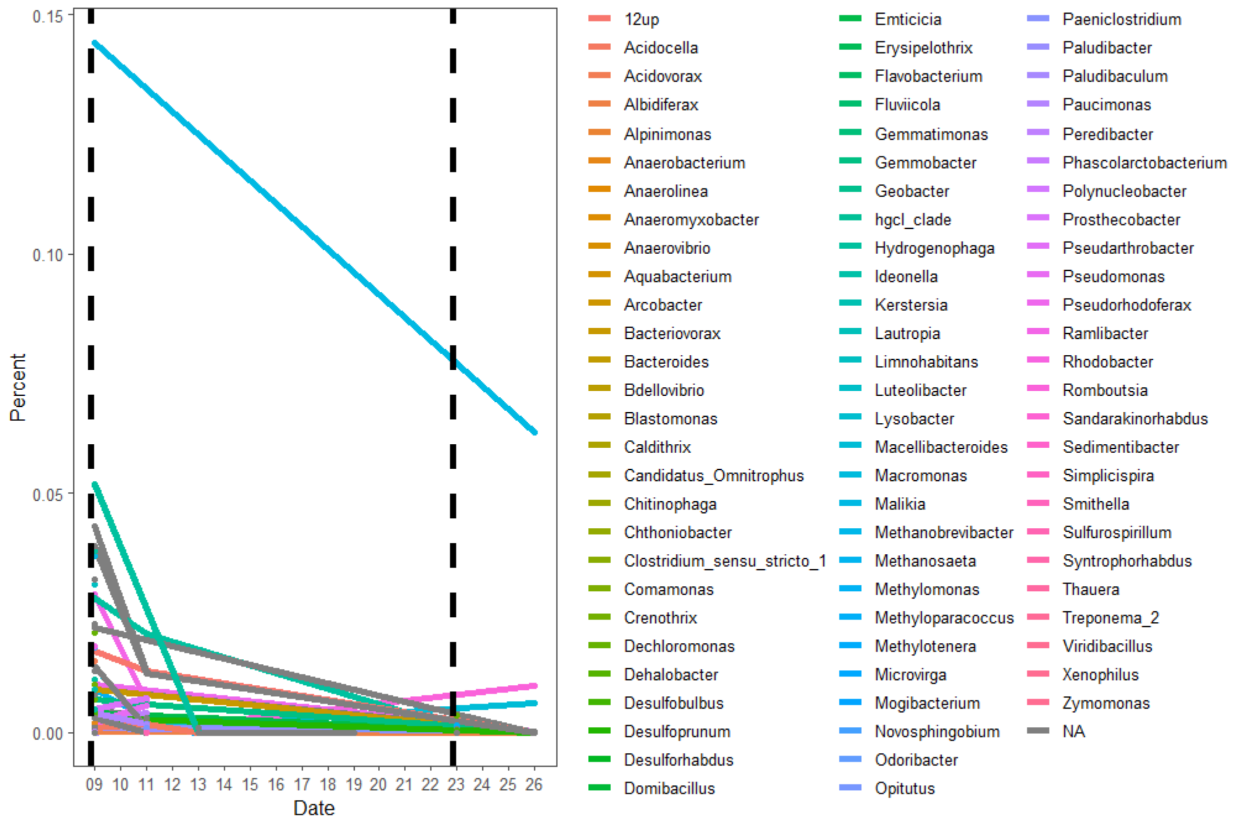

Figure S9: The active microbial taxa (genus) from the bottom of the hippo pool as attributed to upstream by SourceTracker from PRHP during the flushing flows. Dashed lines indicate the flushing flows. The first flushing flow occurred on 8-Aug-17. The second flushing flow occurred on 25-Aug-17.

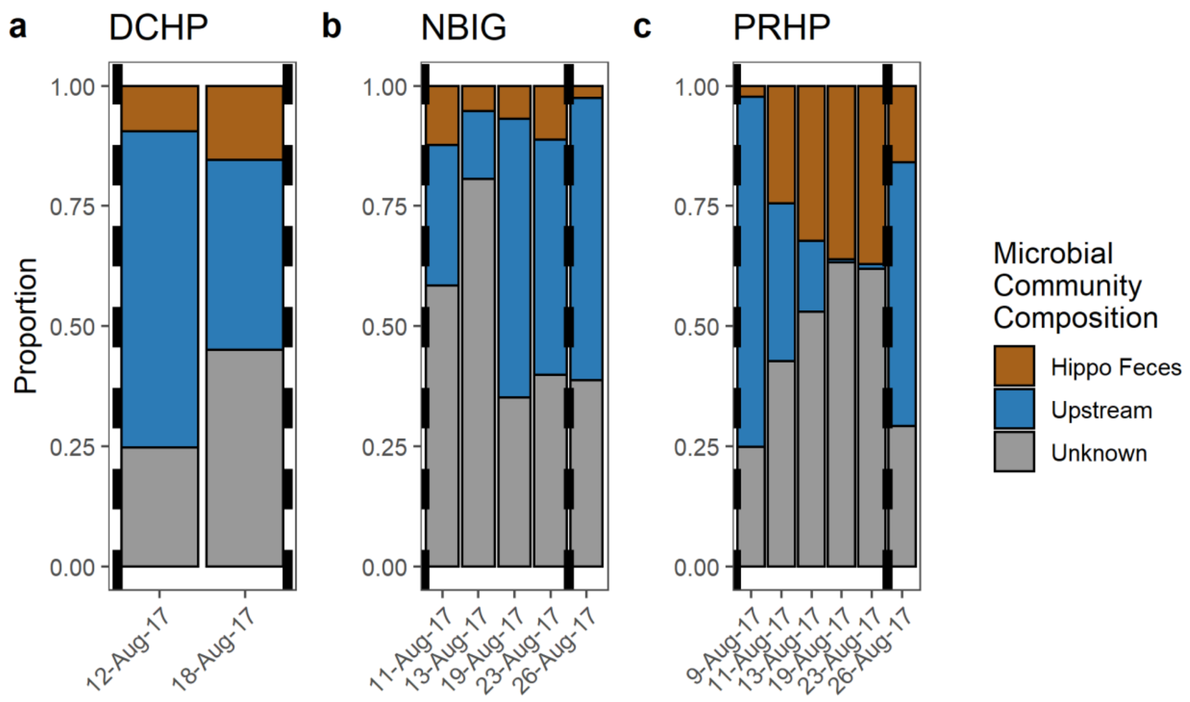

Figure S10: SourceTracker results for the total microbial communities from high-density hippo pools during the flushing flows. Flushing flows are represented by a dashed vertical line. The first flushing flow occurred on 8-Aug-17. The second flushing flow occurred on 25-Aug-17.

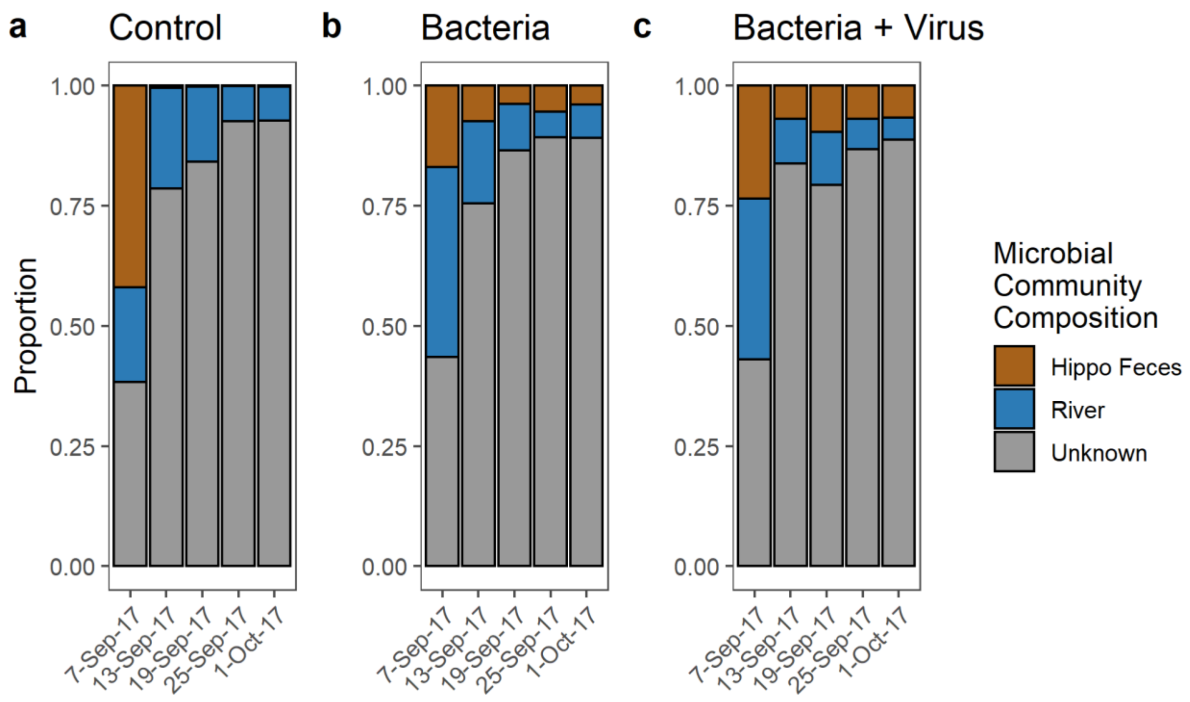

Figure S11: Total microbial community composition from the experimental treatments. SourceTracker results for **(a)** Control (sterilized hippo feces only) **(b)** Hippo feces plus added bacteria, no viruses and **(c)** Hippo feces plus added bacteria and viruses.

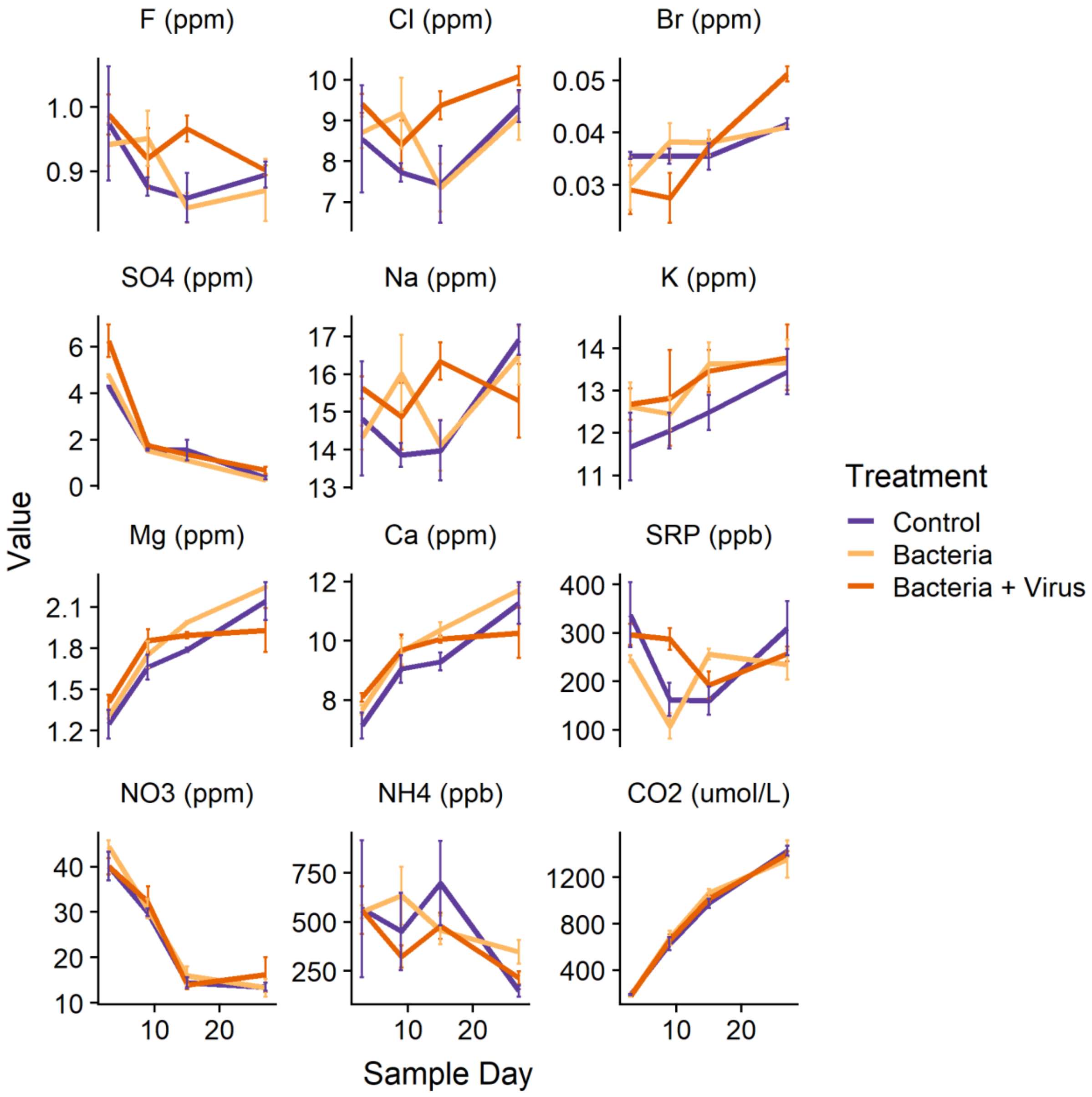

Figure S12: Additional biogeochemical variables from the experiment. Mean concentrations (N=3) and standard deviations as error bars

Table S1: Microbial taxa overlap between the bottom of hippo pools and the hippo gut.

Phylum	Class	Order	Family	Genus	Overlap
Firmicutes	Bacilli	Bacillales	Planococcaceae	NA	16.86%
Bacteroidetes	Bacteroidia	Bacteroidales	Porphyromonadaceae	Macellibacteroides	15.63%
Firmicutes	Erysipelotrichia	Erysipelotrichales	Erysipelotrichaceae	Candidatus_Stoquefichus	8.35%
Tenericutes	Mollicutes	Anaeroplasmatales	Anaeroplasmataceae	Anaeroplasma	6.14%
Firmicutes	Bacilli	Bacillales	Planococcaceae	NA	3.59%
Bacteroidetes	Bacteroidia	Bacteroidales	Prevotellaceae	Prevotellaceae_YAB2003_group	3.51%
Tenericutes	Mollicutes	Anaeroplasmatales	Anaeroplasmataceae	Anaeroplasma	3.40%
Firmicutes	Clostridia	Clostridiales	Lachnospiraceae	NA	3.27%
Bacteroidetes	Bacteroidia	Bacteroidales	Rikenellaceae	dgA-11_gut_group	2.65%
Firmicutes	Clostridia	Clostridiales	Lachnospiraceae	Lachnospiraceae_AC2044_group	2.52%
Bacteroidetes	Bacteroidia	Bacteroidales	Prevotellaceae	Prevotella_7	2.30%
Firmicutes	Clostridia	Clostridiales	Ruminococcaceae	Ruminococcaceae_UCG-010	2.23%
Firmicutes	Clostridia	Clostridiales	Family_XI	Sedimentibacter	2.11%
Bacteroidetes	Bacteroidia	Bacteroidales	Bacteroidaceae	Bacteroides	2.04%
Bacteroidetes	Bacteroidia	Bacteroidales	Prevotellaceae	Prevotellaceae_UCG-001	1.89%
Bacteroidetes	Bacteroidia	Bacteroidales	Prevotellaceae	Prevotella_7	1.86%
Proteobacteria	Gammaproteobacteria	Aeromonadales	Succinivibrionaceae	NA	1.66%
Firmicutes	Clostridia	Clostridiales	Clostridiaceae_1	Clostridium_sensu_stricto_1	1.39%
Firmicutes	Clostridia	Clostridiales	Ruminococcaceae	Ruminococcaceae_UCG-010	1.31%
Firmicutes	Erysipelotrichia	Erysipelotrichales	Erysipelotrichaceae	NA	1.26%
Bacteroidetes	Bacteroidia	Bacteroidales	Prevotellaceae	Prevotellaceae_UCG-001	1.14%
Firmicutes	Clostridia	Clostridiales	Ruminococcaceae	Ruminococcaceae_NK4A214_group	1.09%
Firmicutes	Negativicutes	Selenomonadales	Veillonellaceae	Anaerovibrio	0.97%
Firmicutes	Clostridia	Clostridiales	Lachnospiraceae	NA	0.88%
Firmicutes	Erysipelotrichia	Erysipelotrichales	Erysipelotrichaceae	NA	0.84%
Firmicutes	Clostridia	Clostridiales	Lachnospiraceae	NA	0.71%
Verrucomicrobia	WCHB1-41	NA	NA	NA	0.68%

Phylum	Class	Order	Family	Genus	Overlap
Firmicutes	Clostridia	Clostridiales	Lachnospiraceae	NA	0.63%
Firmicutes	Clostridia	Clostridiales	Clostridiaceae_1	Clostridium_sensu_stricto_1	0.60%
Verrucomicrobia	WCHB1-41	NA	NA	NA	0.53%
Bacteroidetes	Bacteroidia	Bacteroidales	NA	NA	0.49%
Firmicutes	Clostridia	Clostridiales	Lachnospiraceae	NA	0.47%
Proteobacteria	Betaproteobacteria	Burkholderiales	Comamonadaceae	NA	0.46%
Firmicutes	Negativicutes	Selenomonadales	Veillonellaceae	Schwartzia	0.42%
Verrucomicrobia	WCHB1-41	NA	NA	NA	0.38%
Firmicutes	Clostridia	Clostridiales	Lachnospiraceae	NA	0.36%
Bacteroidetes	Bacteroidia	Bacteroidales	NA	NA	0.35%
Firmicutes	Clostridia	Clostridiales	Lachnospiraceae	Anaerosporobacter	0.35%
Firmicutes	Erysipelotrichia	Erysipelotrichales	Erysipelotrichaceae	Erysipelatoclostridium	0.34%
Verrucomicrobia	WCHB1-41	NA	NA	NA	0.33%
Proteobacteria	Betaproteobacteria	Burkholderiales	Comamonadaceae	Ottowia	0.27%
Verrucomicrobia	WCHB1-41	NA	NA	NA	0.26%
Verrucomicrobia	WCHB1-41	NA	NA	NA	0.25%
Bacteroidetes	Bacteroidia	Bacteroidales	Prevotellaceae	Prevotella	0.24%
Bacteroidetes	Bacteroidia	Bacteroidales	Prevotellaceae	Prevotellaceae_UCG-003	0.22%
Firmicutes	Clostridia	Clostridiales	Clostridiaceae_1	Fonticella	0.20%
Bacteroidetes	Bacteroidia	Bacteroidales	Bacteroidales_S24-7_group	NA	0.18%
Firmicutes	Negativicutes	Selenomonadales	Veillonellaceae	NA	0.14%
NA	NA	NA	NA	NA	0.13%
Firmicutes	Clostridia	Clostridiales	Family_XIII	Mogibacterium	0.12%
Firmicutes	Clostridia	Clostridiales	Clostridiaceae_1	Oceanirhabdus	0.11%
Planctomycetes	Planctomycetacia	Planctomycetales	Planctomycetaceae	Singulisphaera	0.11%
Firmicutes	Clostridia	Clostridiales	Ruminococcaceae	NA	0.11%
Proteobacteria	Gammaproteobacteria	Pseudomonadales	Pseudomonadaceae	Pseudomonas	0.11%
Bacteroidetes	Bacteroidia	Bacteroidales	Prevotellaceae	Prevotellaceae_UCG-003	0.11%

Phylum	Class	Order	Family	Genus	Overlap
Firmicutes	Clostridia	Clostridiales	Lachnospiraceae	NA	0.11%
Planctomycetes	Planctomycetacia	Planctomycetales	Planctomycetaceae	p-1088-a5_gut_group	0.11%
Proteobacteria	Gammaproteobacteria	Pseudomonadales	Pseudomonadaceae	Pseudomonas	0.09%
Verrucomicrobia	WCHB1-41	NA	NA	NA	0.07%
Firmicutes	Clostridia	Clostridiales	Lachnospiraceae	NA	0.07%
Firmicutes	Clostridia	Clostridiales	Lachnospiraceae	Lachnospiraceae_NC2004_group	0.07%
Proteobacteria	Epsilonproteobacteria	Campylobacterales	Campylobacteraceae	Campylobacter	0.07%
NA	NA	NA	NA	NA	0.07%
Firmicutes	Clostridia	Clostridiales	Lachnospiraceae	Lachnospiraceae_NC2004_group	0.06%
Firmicutes	Negativicutes	Selenomonadales	Veillonellaceae	Selenomonas_1	0.06%
Bacteroidetes	Bacteroidia	Bacteroidales	Prevotellaceae	Prevotellaceae_UCG-003	0.06%
Firmicutes	Negativicutes	Selenomonadales	Veillonellaceae	NA	0.05%
Verrucomicrobia	WCHB1-41	NA	NA	NA	0.05%
Verrucomicrobia	WCHB1-41	NA	NA	NA	0.05%
Proteobacteria	Alphaproteobacteria	Rhodobacterales	Rhodobacteraceae	Defluviimonas	0.05%
Euryarchaeota	Methanobacteria	Methanobacteriales	Methanobacteriaceae	Methanosphaera	0.05%
Firmicutes	Clostridia	Clostridiales	Lachnospiraceae	Lachnospiraceae_UCG-007	0.04%
Firmicutes	Clostridia	Clostridiales	Family_XIII	Mogibacterium	0.04%
Planctomycetes	Planctomycetacia	Planctomycetales	Planctomycetaceae	Singulisphaera	0.04%
Proteobacteria	Alphaproteobacteria	Rhizobiales	Methylobacteriaceae	Methylobacterium	0.04%
Proteobacteria	Gammaproteobacteria	Xanthomonadales	Xanthomonadaceae	Lysobacter	0.03%
Proteobacteria	Epsilonproteobacteria	Campylobacterales	Campylobacteraceae	Arcobacter	0.03%
Verrucomicrobia	WCHB1-41	NA	NA	NA	0.03%
Bacteroidetes	Bacteroidia	Bacteroidales	Bacteroidales_RF16_group	NA	0.03%
Verrucomicrobia	WCHB1-41	NA	NA	NA	0.03%
Elusimicrobia	Elusimicrobia	Elusimicrobiales	Elusimicrobiaceae	Elusimicrobium	0.02%
Proteobacteria	Gammaproteobacteria	Pseudomonadales	Moraxellaceae	Acinetobacter	0.02%
Firmicutes	Clostridia	Clostridiales	Clostridiaceae_1	Clostridium_sensu_stricto_1	0.02%
Firmicutes	Clostridia	Clostridiales	Lachnospiraceae	NA	0.02%

Phylum	Class	Order	Family	Genus	Overlap
Proteobacteria	Alphaproteobacteria	Caulobacterales	Caulobacteraceae	Phenylobacterium	0.01%
Bacteroidetes	Bacteroidia	Bacteroidales	Porphyromonadaceae	NA	0.01%
Firmicutes	Clostridia	Clostridiales	Peptococcaceae	Peptococcus	0.01%

Table S2: Site locations for the longitudinal transects sampled down the Mara and Talek rivers in 2017.

DATE	TIME	Site	Location (decimal degrees)	
			Latitude	Longitude
11/8/2017	9:58:00	Mara 1	-1.05634	35.23141
11/8/2017	10:42:00	Mara 2	-1.09578	35.20228
11/8/2017	11:30:00	Mara 3	-1.13743	35.13311
11/8/2017	12:16:00	Mara 4	-1.22289	35.03641
11/8/2017	13:20:00	Mara 5	-1.31761	35.02211
11/8/2017	14:03:00	Mara 6	-1.39484	35.03452
11/8/2017	14:34:00	Mara 7	-1.43513	35.06147
11/8/2017	14:56:00	Mara 8	-1.46878	35.03342
11/8/2017	15:16:00	Mara 9	-1.50692	35.02615
11/8/2017	15:44:00	Mara 10	-1.54626	35.01909
11/4/2017	10:41:00	Talek 1	-1.47067	35.30169
11/4/2017	11:09:00	Talek 2	-1.46269	35.26452
11/4/2017	11:26:00	Talek 3	-1.45984	35.24873
11/4/2017	12:12:00	Talek 4	-1.44331	35.20824
11/4/2017	14:00:00	Talek 5	1.42635	35.17826
11/4/2017	14:15:00	Talek 6	-1.41312	35.1353
11/4/2017	14:38:00	Talek 7	-1.41661	35.09698
11/4/2017	15:05:00	Talek 8	-1.42281	35.08352

Supplementary Text

Preliminary experiment to test the efficacy of sterilizing hippo feces in a pressure cooker

We used sterilized hippo feces as an organic matter substrate for our bottle experiment. Because our experiment took place at our remote field camp in the Maasai Mara National Reserve, alternatives to an autoclave were explored as a method to sterilize fresh hippo feces. We tested three methods of sterilization that we would have access to at our field site; a pressure cooker, drying in the sun and drying in an oven. We used 5 replicates of each of the three treatments in addition to also using an autoclave. After attempting sterilization using the different methods, we tested biological activity by measuring dissolved oxygen over time after submerging the sterilized material in water.

Setup

Autoclave - We placed 15 mg of fresh hippo feces into 5 acid washed beakers. The 5 beakers of fresh hippo feces were then placed inside of an autoclave bag and loosely sealed. The autoclave was run one hour on the standard cycle.

Pressure Cooker – We placed 15 mg of fresh hippos feces into a pressure cooker (6-quart aluminium, National Presto Industries, Inc., Eau Claire, WI, USA) with a small amount of water. We applied heat to each of the five replicates in the pressure cooker and attained maximum pressure for 5 minutes.

Oven Dried – We placed five replicates of 15 mg of fresh hippo feces in a drying oven at 60C for 24 hours.

Sun Dried – We placed five replicates of 15 mg of fresh hippo feces on a sheet of aluminium foil in the sun for 6 hours on a cloudy day.

We then took each replicate from each treatment and spiked it into 200 mL of oxygenated nanopure water (8.25 mg/L). We measured dissolved oxygen in each treatment replicate after the addition at 15 minutes, 1020 minutes and 2460 minutes. We also spiked five replicates of 15mg of fresh hippo feces into 200 mL of oxygenated water.

Results/Conclusions

Autoclaved hippo feces and pressure-cooked hippo feces responded similarly (Fig. S13). There was a slow drop in dissolved oxygen over the experiment that likely indicates a similar amount of biological activity between autoclaving and pressure cooking. Oven drying and sun drying also had similar results, but were less effective than autoclaving or pressure cooking. Dissolved oxygen reached zero mg/L by 2460 minutes in every treatment (except for the water-only control). The water control showed no biological activity (no drop in dissolved oxygen). Untreated fresh hippo feces showed the quickest drop in dissolved oxygen.

This data does not accurately represent the slope of the oxygen decline in the hippo feces, sun dried or oven dried treatments. The dissolved oxygen was likely depleted in the hippo feces

treatment within approximately 60 minutes. The dissolved oxygen was likely depleted in the sun dried and oven dried treatments within 120 minutes.

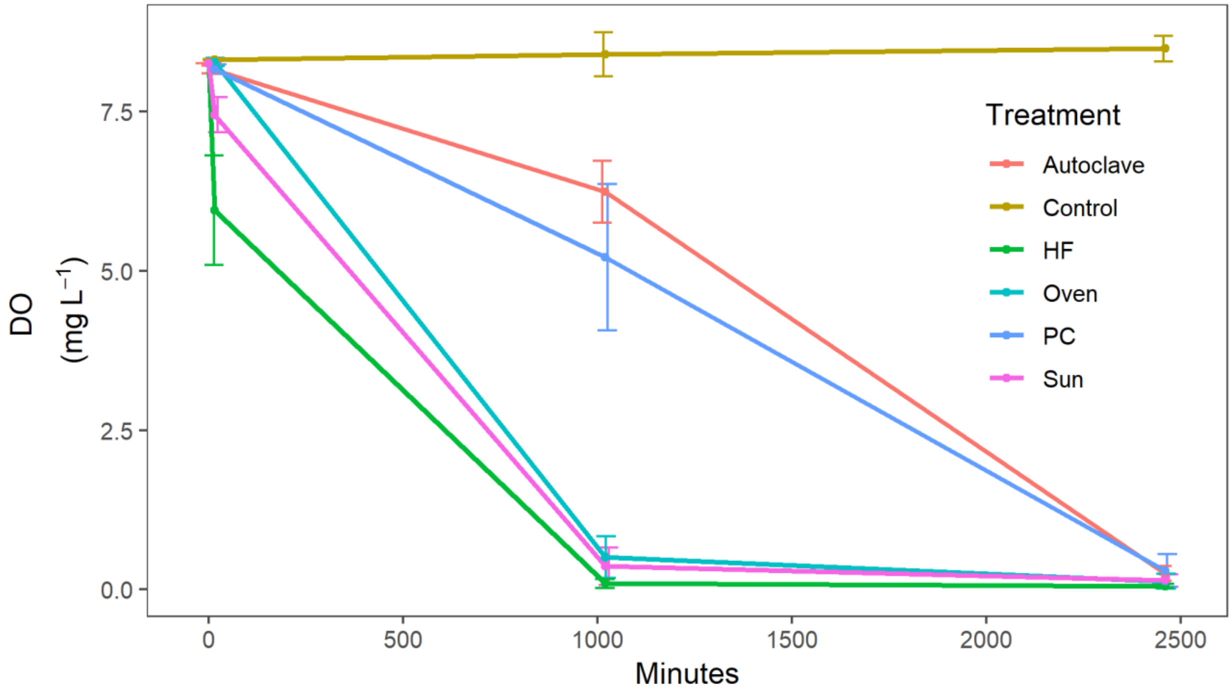

Figure S13: Dissolved oxygen measured over time for the three treatments (oven dried, sun dried, pressure cooker) as well as fresh hippo feces, autoclaved hippo feces and a control (water blank).

Experiment to test if a short-term UV treatment affects DOM quality

UV light can alter DOC quality and affect biogeochemical cycling. To determine if DOC quality is altered after a short 2-minute UV light treatment with a Steripen (Katadyn Group, Switzerland), we conducted a small experiment.

The original hippo feces liquid used in the experiment had a DOC concentration of 247 mg/L. We recreated a similar solution in the laboratory at Yale University with hippo feces from the Milwaukee County Zoo. We subjected half of that solution to a 2-minute UV light treatment and left the other half as a control. We then did dilutions using MQ water down to approximately 2 mg/L of DOC for the treatment and control. DOC quality analysis was done on an Aqualog (Horiba Scientific, Kyoto, Japan). We had two replicates of the control and of the treatment.

In the UV light treatment, we degraded aromaticity (SUVA) between 0.7 to 1.5% of the total DOM pool within each bottle and do not believe that this would cause any difference between treatments.
